# Risk and protective factors for postoperative anastomotic leakage in esophageal and gastrointestinal surgery: an umbrella review of meta-analyses and systematic reviews

**DOI:** 10.1097/JS9.0000000000003308

**Published:** 2025-09-19

**Authors:** Xianrong Bao, Keqian Yi, Jibin Cheng, Yang Shen, Song Cao, Biao Hu, Xingzhi Wang, Pengwei Su, Yijun Li, Qingwen Xu, Pengyuan Xu

**Affiliations:** a Department of Gastrointestinal Surgery, Second Affiliated Hospital of Kunming Medical University, Kunming, China; b The Second Hospital and Clinical Medical School, Lanzhou University, Lanzhou, China; c Second Faculty of Clinical Medicine, Kunming Medical University, Kunming, China; d Department of Gastroenterology, Second Affiliated Hospital of Kunming Medical University, Kunming, China; e Department of Radiation Oncology, Yunnan Cancer Hospital, The Third Affiliated Hospital of Kunming Medical University, Kunming, China

**Keywords:** anastomotic leakage, meta-analysis, umbrella review

## Abstract

**Background and Objective:**

Anastomotic leakage (AL) is a common and serious complication in gastrointestinal surgery, which significantly affects patient recovery and long-term prognosis. This umbrella review aims to summarize the risk and protective factors for AL after gastric, esophageal, and colorectal cancer surgeries, and to provide a comprehensive evaluation of the quality of existing literature, offering guidance for clinical practice.

**Methods:**

A systematic search was conducted to identify eligible meta-analyses. For each included study, we recalculated and assessed the risk estimates, heterogeneity, small-study effects, excess significance testing, and publication bias. Additionally, we considered the quality of the studies and graded the evidence.

**Results:**

A total of 173 potential associations were included. The analysis revealed that ASA scores (3–4), male gender, diabetes, hypertension, and chronic kidney disease were significantly associated with an increased risk of AL. Preoperative mechanical bowel preparation combined with oral antibiotics significantly reduced the incidence of AL. Intraoperative use of collagen or fibrin-based sealants, indocyanine green (ICG) fluorescence imaging, flexible endoscopic examination, and leak tests were all significantly associated with reduced AL risk. The use of nonsteroidal anti-inflammatory drugs (NSAIDs) was linked to an increased risk of AL. In rectal cancer surgeries, low-anterior resection was associated with a significantly higher risk of AL. In esophageal cancer surgeries, the incidence of AL was higher after transthoracic anastomosis than after cervical anastomosis, although the severity of complications associated with cervical anastomoses was lower.

**Conclusion:**

AL remains a major challenge in gastrointestinal surgery, and involves multiple risk factors. Optimizing perioperative management, refining intraoperative techniques, and judicious use of antibiotics and NSAIDs can significantly reduce the risk of AL. Future research should focus on high-quality, large-sample, multicenter studies to explore more effective prevention and treatment strategies.

**HIGHLIGHTS**

- This umbrella review provides the first comprehensive synthesis of risk and protective factors associated with anastomotic leakage (AL) after gastrointestinal surgery.
- A total of 14 risk factors and 10 protective factors for AL were identified and systematically evaluated.
- Evidence strength was graded using rigorous criteria for bias, heterogeneity, and methodological quality.
- NSAIDs, particularly nonselective agents such as diclofenac, were strongly associated with increased AL risk.
- Protective strategies including ICG fluorescence imaging, combined oral antibiotics with mechanical bowel preparation, and leak testing methods were supported by robust evidence.
- Our findings offer critical insights to inform preoperative planning, intraoperative decision-making, and postoperative management to reduce AL incidence.

## Introduction

Anastomotic leakage (AL) is a serious complication following gastrointestinal surgery, which can lead to infections, peritonitis, abscesses, and other complications^[1]^. It poses a threat to patients’ lives and increases hospital stay, reoperation risk, and financial burden on the patients^[2]^. Despite advancements in medical technology, its incidence remains high, ranging from 10% to 20%^[3]^. This challenge is related to multiple factors, including patient-specific characteristics, preoperative preparation, surgical complexity, and postoperative care, making it a critical issue for surgeons to address.

Numerous studies have identified various risk factors that affect anastomotic healing, including patient age, nutritional status, and comorbidities such as diabetes and cardiovascular disease^[4–6]^. Additionally, intraoperative factors such as surgical technique, anastomotic method, prolonged operative time, and intraoperative blood loss have been shown to significantly influence AL risk^[7–10]^. To reduce the incidence of AL, a variety of protective strategies have been adopted in clinical practice, including preoperative bowel preparation, prophylactic antibiotics, ischemic conditioning, intraoperative techniques like the use of staplers, indocyanine green (ICG) fluorescence imaging, reinforcement materials, and omental wrapping, as well as postoperative nutritional and pain management strategies^[11–22]^.

Although many systematic reviews and meta-analyses have explored the risk and protective factors for AL following gastrointestinal surgery, there remain several limitations. Existing evidence shows methodological variability, high heterogeneity, and inconsistent conclusions. For instance, the impact of nonsteroidal anti-inflammatory drugs (NSAIDs) on AL risk remains controversial, with conflicting results across different studies^[21,23]^. Similarly, the clinical efficacy and applicability of several protective strategies remain under debate. These observations demonstrate that some studies on influential factors for anastomotic leakage have yielded rather limited and inconsistent conclusions, providing only limited clinical guidance due to the lack of comprehensive analyses. Therefore, the comprehensive and methodologically rigorous synthesis and reanalysis of the existing studies are urgently needed to summarize and evaluate the reliability of current evidence.

This study represents the first umbrella review to systematically synthesize and evaluate risk and protective factors for AL following esophageal, gastric, and colorectal surgeries. Without the assistance of artificial intelligence (AI) as outlined in the TITAN 2025 guidelines^[24]^, using strict criteria for bias, heterogeneity and methodological quality, this review not only identifies high-confidence factors but also highlights unresolved controversies and gaps in the literature. The findings aim to inform and support clinical decision-making in preoperative assessment, intraoperative management, and postoperative care, ultimately contributing to a reduction in AL incidence and improved patient outcomes.

## Methods

This study was registered with PROSPERO (Registration Number: anonymous) (https://www.crd.york.ac.uk/PROSPERO) and was conducted and reported in strict accordance with the Preferred Reporting Items for Systematic Reviews and Meta-Analyses (PRISMA) guidelines and the outlined methodology^[25,26]^.

## Search strategy

We systematically searched the PubMed, Embase, Cochrane Library, and Web of Science databases up to November 2024. The search strategy included a combination of Medical Subject Headings (MeSH) terms and their synonyms, such as “anastomotic leak,” “meta-analysis,” and “rectal cancer,” following the Scottish Intercollegiate Guidelines Network’s guidance for literature search^[27]^. Additionally, we screened recently published articles in high-impact journals to ensure that no significant studies related to anastomotic leaks were overlooked. The entire process was independently conducted by two researchers, who also verified the accuracy and reproducibility of the search strategy.

## Eligibility criteria

Following the database search, all meta-analyses or systematic reviews exploring interventions or exposure factors related to gastrointestinal anastomotic leaks were thoroughly reviewed and assessed for usability. To ensure high-quality evidence, we prioritized the most recently published studies or those incorporating the largest number of randomized controlled trials (RCTs) and cohort studies. Duplicate publications or studies with significant overlap with others were excluded, along with animal studies, *in vitro* research, conference abstracts, editorials, letters, non-English publications, and studies with incomplete data.

The following studies were excluded: meta-analyses that did not formally present associations between any factors and the outcomes of interest; meta-analyses that did not provide formal risk estimates; simple RCTs or clinical trials rather than secondary research based on them; studies focusing on pediatric or adolescent populations; and non-English articles.

## Data extraction

Two researchers independently extracted the following data: study details (authors, year, journal), study design, patient characteristics, information on the intervention and control groups, incidence of anastomotic leak (number of cases and total sample size), and effect estimates (RR or OR with 95% confidence interval). Additionally, *P*-values, I^2^ statistic, details of subgroup analysis, assessment of publication bias, and raw data for overall effect estimates were recorded. Double extraction ensured consistency, and ambiguous variables were documented and resolved through further team discussions to reach consensus.

## Data analysis

### Calculation and reanalysis of the overall effect estimates, heterogeneity, and 95% prediction intervals

After extracting the raw data of the overall effect estimates from the included meta-analyses or systematic reviews, we recalculated and reanalyzed the current 95% confidence intervals and *I*^2^ statistics to assess heterogeneity^[26,28,29]^. Based on the heterogeneity results, the overall effect estimates corresponding to the appropriate effect model were presented in the primary analysis. Additionally, 95% prediction intervals were introduced to account for the uncertainty of individual data points^[28,30]^.

For meta-analyses that included both RCTs and observational studies and conducted stratification based on study design, we selected the subgroup analysis results based on RCTs for data extraction and reanalysis. All data extractions were independently performed by six authors and independently verified by four authors. Any discrepancies were resolved through discussion.

### Assessment of publication bias, small-study effects, and excessive significance bias

We calculated publication bias using Egger’s regression test for all reanalyses, determining the Egger’s *P*-value and the point estimate of the largest study (smallest standard error) to evaluate the presence of small-study effects^[31–33]^. To assess excessive significance bias, we applied the Proportion of Statistical Significance Test (PSST) and the Enhanced Statistical Significance Test (ESST). A result of “0” for both PSST and ESST indicated the absence of excessive significance bias. Additionally, we incorporated the standard error (SE) of each study, along with the mean and variance of the true effect distribution in the meta-analysis, to further refine the assessment of potential bias and the reliability of effect estimates. This approach enhanced the scientific rigor and reproducibility of the analytical results^[34–36]^.

### Assessment of the quality of evidence and methodological quality of the included studies

Following published guidelines, the reliability of evidence for the included factors was categorized into five levels based on the following criteria: the number of cases, statistical significance, and heterogeneity (Supplementary Digital Content Table S1, available at: http://links.lww.com/JS9/F138, Supplementary Digital Content Fig. S1, available at: http://links.lww.com/JS9/F143)^[37–42]^. Additionally, the AMSTAR 2 tool was used to systematically evaluate the methodological quality of the included systematic reviews and meta-analyses, classifying them into four distinct levels^[43]^.

## Results

### Characteristics of meta-analyses

**Figure 1.**
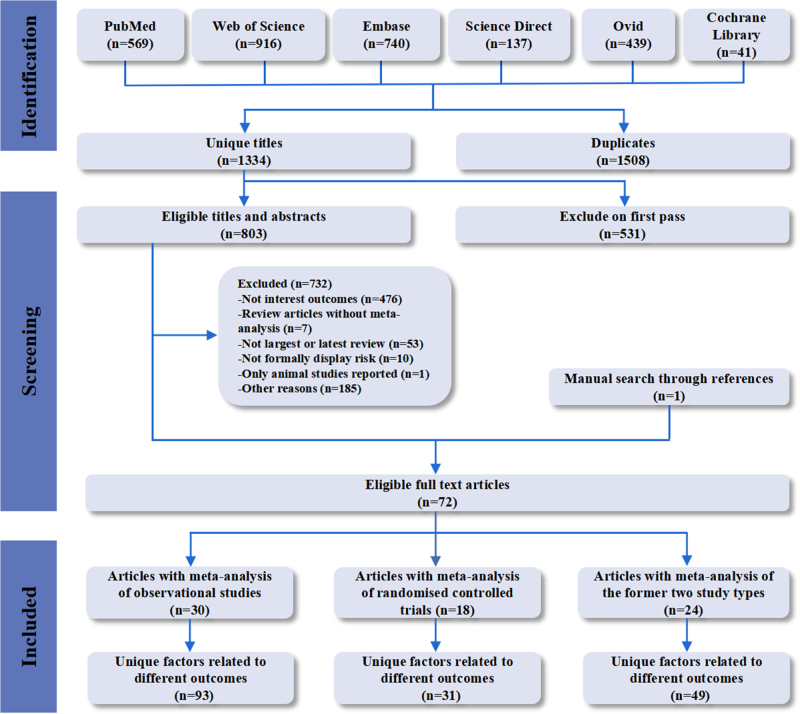
Summary of the evidence search and selection process.

Figure 1 illustrates the process of literature search and selection^[44]^. A total of 2842 unique studies were identified through database search. After further deduplication, application of inclusion and exclusion criteria, and full-text review, a total of 72 studies^[4-22,45–97]^ were included, resulting in 173 risk associations between various factors and the target outcomes.

### Quality assessment of meta-analyses

Based on the AMSTAR-2 tool, the quality assessment of the 72 included studies revealed that most had low quality (Supplementary Digital Content Table S5, available at: http://links.lww.com/JS9/F142)^[43]^. Only one study was rated as “high quality”^[14]^, while 58 and 13 studies were rated as “low quality” and “very low quality,” respectively. The most commonly missing items were Item 10 (source of funding of primary studies), followed by Item 7 (a list of excluded studies and justifications for the exclusions) and Item 3 (an explanation of the selection of study designs).

### Grading of evidence

Among the included 173 exposure or intervention factors, 56 investigated the association with esophageal cancer, 23 with gastric cancer, and 94 with colorectal cancer. We identified 73 factors with beneficial significant associations and 100 factors with harmful significant associations. Using the evidence grading criteria, we found that only 2 associations reached Class I (1.2%), 16 reached Class II (9.4%), 6 reached Class III (3.5%), and 49 reached Class IV (28.7%).

#### Esophageal cancer

**Figure 2.**
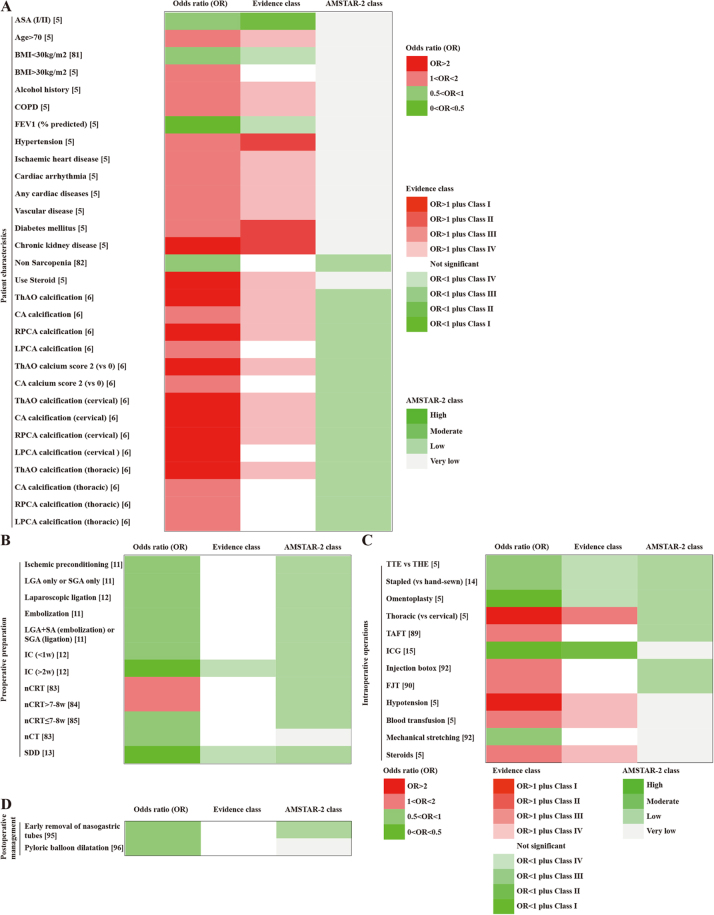
Summary of risk estimates for the association between esophageal cancer (EC) postoperative anastomotic leak and various factors.

**Patient characteristics** (Fig. 2A and Supplementary Digital Content Table S2, available at: http://links.lww.com/JS9/F139). A meta-analysis including 12 observational studies investigated the association between the American Society of Anesthesiologists (ASA) classification and the occurrence of anastomotic leaks^[5]^. The study showed that patients with preoperative ASA score I or II had a significantly lower risk of anastomotic leaks (OR, 0.501; 0.380–0.660) (Class II). The study also revealed significant associations between an increased risk of anastomotic leaks and comorbidities such as hypertension (OR, 1.467; 1.192–1.806) (Class II), diabetes (OR, 1.639; 1.407–1.909) (Class II), and chronic kidney disease (OR, 3.103; 2.141–4.497) (Class II)^[5]^.

(A) Overall EC: patient characteristics.

(1) Evidence class: Darker green represents stronger protective effects, while darker red indicates a higher risk effects. (2) AMSTAR-2 class: Darker green denotes higher methodological quality based on AMSTAR-2 assessment.

ASA, American Society of Anaesthesiologists; BMI, body mass index; COPD, chronic obstructive pulmonary disease; FEV1, forced expiratory volume in 1 second; ThAO, thoracic aorta; CA, celiac axis; RPCA, right post-celiac axis; LPCA, left post-celiac axis

(B) Overall EC: perioperative preparation.

(C) Overall EC: intraoperative procedures.

(D) Overall EC: postoperative treatment.

***Explanatory note:*** (1) Evidence class: Darker green represents stronger protective effects, while darker red indicates a higher risk effects. (2) AMSTAR-2 class: Darker green denotes higher methodological quality based on AMSTAR-2 assessment.

LGA, left gastric artery; SGA, short gastric arteries; SA, splenic artery; IC, ischemic conditioning; nCRT, neoadjuvant chemoradiotherapy; nCT, neoadjuvant chemotherapy; SDD, selective decontamination of the digestive tract; TTE, transthoracic esophagectomy; THE, transhiatal esophagectomy; TAFT, transanastomotic feeding tube; ICG, indocyanine green; FJT, feeding jejunostomy tube; AMSTAR 2, a measurement tool to assess systematic reviews.

**Preoperative preparation** (Fig. 2B and Supplementary Digital Content Table S2, available at: http://links.lww.com/JS9/F139). The available studies showed that an interval of more than 2 weeks between ischemic conditioning (IC) and anastomosis (OR, 0.334; 0.166–0.670) (Class IV)^[12]^ and preoperative selective gastrointestinal decontamination (OR, 0.388; 0.188–0.801) (Class IV)^[13]^ were able to significantly reduce the risk of anastomotic leaks.

**Intraoperative procedures** (Fig. 2C and Supplementary Digital Content Table S2, available at: http://links.lww.com/JS9/F139). A meta-analysis including nine observational studies investigated the role of ICG fluorescence imaging in reducing the risk of anastomotic leaks after minimally invasive Ivor Lewis esophagectomy^[15]^. The study revealed that ICG fluorescence imaging significantly reduced the risk of anastomotic leaks (OR, 0.252; 0.152–0.418) (Class II). Another meta-analysis on surgical techniques showed that the risk of anastomotic leaks was significantly higher after thoracic anastomosis than after cervical anastomosis (OR, 2.084; 1.561–2.781) (Class III)^[5]^.

#### Gastric cancer

**Figure 3.**
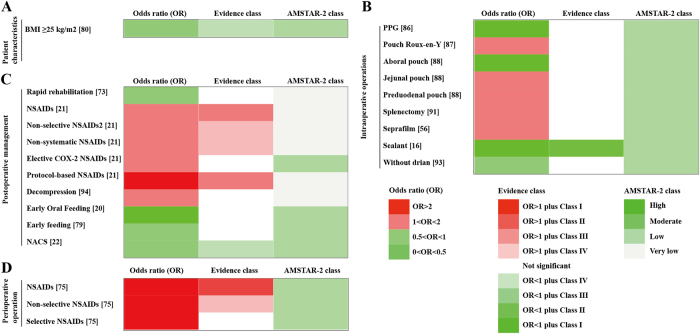
Summary of risk estimates for the association between postoperative anastomotic leak and various factors in gastric cancer (GC) surgery.

**Patient characteristics** (Fig. 3A and Supplementary Digital Content Table S3, available at: http://links.lww.com/JS9/F140). A body mass index (BMI) ≥ 25 kg/m^2^ was found to be significantly associated with an increased risk of anastomotic leaks (OR, 0.565; 0.443–0.722) (Class IV)^[80]^.

(A) Overall GC: patient characteristics.

(B) Overall GC: intraoperative procedures.

(C) Overall GC: postoperative treatment.

(D) Overall GC: perioperative preparation.

(1) Evidence class: Darker green represents stronger protective effects, while darker red indicates a higher risk effects. (2) AMSTAR-2 class: Darker green denotes higher methodological quality based on AMSTAR-2 assessment.

BMI, body mass index; PPG, pylorus-preserving gastrectomy; NSAIDs, non-steroidal anti-inflammatory drugs; NACS, neoadjuvant chemotherapy followed by surgery; AMSTAR 2, a measurement tool to assess systematic reviews.

**Intraoperative procedures** (Fig. 3B and Supplementary Digital Content Table S3, available at: http://links.lww.com/JS9/F140). A meta-analysis including five RCTs and nine observational studies investigated the use of coatings or reinforcement materials at the anastomosis during surgery and their relationship with postoperative anastomotic leaks^[16]^. The study revealed that the use of coatings or reinforcement materials at the anastomosis significantly reduced the risk of postoperative anastomotic leaks (OR, 0.374; 0.271–0.517) (Class II).

**Postoperative treatment** (Fig. 3C and Supplementary Digital Content Table S3, available at: http://links.lww.com/JS9/F140). A meta-analysis showed that postoperative use of NSAIDs significantly increased the risk of anastomotic leaks (OR, 1.688; 1.278–2.230) (Class III), and the use of protocol-based NSAIDs further significantly increased the risk of anastomotic leaks (OR, 4.507; 2.798–7.260) (Class III)^[21]^.

**Perioperative preparation** (Fig. 3D and Supplementary Digital Content Table S3, available at: http://links.lww.com/JS9/F140). A meta-analysis examining the relationship between perioperative use of nonsteroidal anti-inflammatory drugs (NSAIDs) and anastomotic leaks^[75]^ demonstrated that nonselective NSAIDs significantly increased the risk of anastomotic leaks (OR, 3.018; 2.155–4.227) (Class II), whereas selective NSAIDs had no significant effect.

#### Colorectal cancer

**Figure 4.**
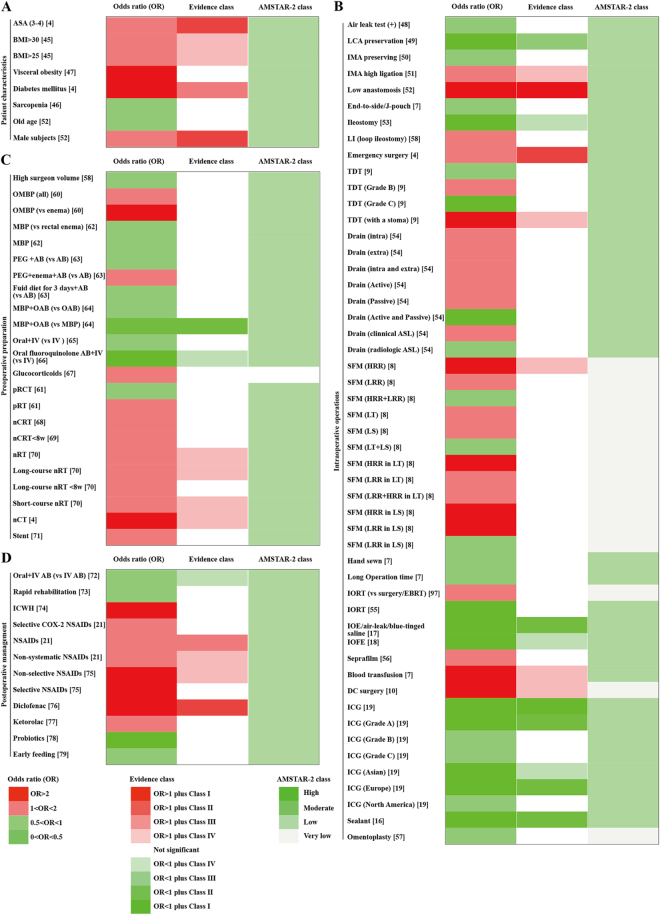
Summary of risk estimates for the association between anastomotic leak and various factors after colorectal cancer surgery.

**Patient characteristics** (Fig. 4A and Supplementary Digital Content Table S4, available at: http://links.lww.com/JS9/F141). A meta-analysis on preoperative risk factors for anastomotic leaks after colorectal cancer resection revealed significant associations between an increased risk of anastomotic leaks and ASA (grade 3 or 4) (OR, 1.762; 1.377–2.254) (Class II) and diabetes (OR, 2.138; 1.490–3.067) (Class III)^[4]^. Additionally, compared with female patients, male patients had a significantly higher risk of anastomotic leaks (OR, 1.478; 1.365–1.601) (Class II)^[4]^.

(A) Overall CRC: patient characteristics.

(B) Overall CRC: perioperative preparation.

(C) Overall CRC: intraoperative procedures.

(D) Overall CRC: postoperative treatment.

(1) Evidence class: Darker green represents stronger protective effects, while darker red indicates a higher risk effects. (2) AMSTAR-2 class: Darker green denotes higher methodological quality based on AMSTAR-2 assessment.

ASA, American Society of Anaesthesiologists; BMI, body mass index; LCA, left colic artery; IMA, inferior mesenteric artery; LI, loop ileostomy; TDT, transanal drainage tube; SFM, splenic flexure mobilization; HRR, high rectal resection; LRR, low rectal resection; LT, laparotomic; LS, laparoscopic; IORT, intraoperative radiotherapy; EBRT, external beam radiotherapy; IOE, intraoperative endoscopy; IOFE, intra-operative flexible endoscopy; Seprafilm, hyaluronate-carboxy-methylcellulose-based membrane; DC, damage control; ICG, indocyanine green; OMBP, oral mechanical bowel preparation; MBP, mechanical bowel preparation; PEG, polyethylene glycol; AB, antibiotics; OAB, oral antibiotics; IV, intravenous; pRCT, preoperative chemoradiotherapy; pRT, preoperative radiotherapy; nCRT, neoadjuvant chemoradiotherapy; nRT, neoadjuvant radiotherapy; nCT, neoadjuvant chemotherapy; ICWH, intraperitoneal chemotherapy without hyperthermia; NSAIDs, non-steroidal anti-inflammatory drugs; AMSTAR 2, a measurement tool to assess systematic reviews.

**Intraoperative procedures** (Fig. 4B and Supplementary Digital Content Table S4, available at: http://links.lww.com/JS9/F141). A meta-analysis consisting of observational studies revealed that, compared with high rectal anastomosis (defined as a low anterior resection for upper rectal cancer (12–16 cm), with colorectal anastomosis below the anterior peritoneal reflection but proximal to the anorectal junction), low rectal anastomosis (defined by most studies as an anastomosis 5 cm or less from the anal verge^[51]^, or as an ultralow anterior resection or coloanal pull-through anastomosis with total mesorectal excision for mid- (8–12 cm) and distal (< 8 cm) rectal cancers, with the coloanal anastomosis at or below the anorectal junction) significantly increased the risk of anastomotic leaks (OR, 3.265; 2.314–4.608) (Class I)^[52]^.

A meta-analysis including three RCTs and 19 observational studies demonstrated that ICG fluorescence angiography significantly reduced the risk of anastomotic leaks following radical surgery for right colon cancer (OR, 0.350; 0.274–0.448) (Class I). Subgroup analysis revealed that ICG fluorescence angiography significantly reduced the risk of anastomotic leaks in grade A leaks (subclinical leakage or imaging leakage) (OR, 0.217; 0.117–0.402) (Class II), in European populations (OR, 0.320; 0.218–0.467) (Class II), and in Asian populations (OR, 0.379; 0.178–0.804) (Class IV). However, this technique showed no statistically significant association with the risk of anastomotic leaks in North American populations or in grade B and C anastomotic leaks^[19]^.

Our study showed that emergency surgery was significantly associated with an increased risk of anastomotic leaks following radical surgery for colorectal cancer (OR, 1.675; 1.285–2.184) (Class II)^[4]^.

A meta-analysis including 2 RCTs and 10 observational studies examined the use of flexible endoscopy, leak tests via Foley catheter, or methylene blue tests during surgery. It was found that these methods for intraoperative anastomosis integrity testing and perfusion assessment significantly reduced the risk of anastomotic leaks following surgery for colorectal diseases (OR, 0.494; 0.362–0.676) (Class II)^[17]^.

Similar to the results in the field of gastric surgery, the use of coatings or reinforcement materials at the anastomosis significantly reduced the risk of anastomotic leaks after colorectal surgery (OR, 0.374; 0.271–0.517) (Class II)^[16]^. Additionally, intraoperative preservation of the left colic artery (LCA) significantly reduced the risk of anastomotic leaks following radical surgery for right colon cancer (OR, 0.430; 0.289–0.640) (Class III)^[49]^.

**Preoperative preparation** (Fig. 4C and Supplementary Digital Content Table S4, available at: http://links.lww.com/JS9/F141). A meta-analysis including 26 RCTs and 9 observational studies investigated the use of oral antibiotics in elective colorectal surgery^[64]^. The study showed that compared with mechanical bowel preparation alone, the use of combined oral antibiotics significantly reduced the risk of anastomotic leaks (OR, 0.454; 0.421–0.490) (Class II).

**Postoperative treatment** (Fig. 4D and Supplementary Digital Content Table S4, available at: http://links.lww.com/JS9/F141). A meta-analysis including 6 RCTs and 18 observational studies investigated the relationship between the use of NSAIDs and anastomotic leaks following surgery for colorectal diseases^[21]^. The study revealed a significant association between the use of NSAIDs and an increased risk of anastomotic leaks (OR, 1.688; 1.278–2.230) (Class III). The study also demonstrated that the use of diclofenac significantly increased the risk of anastomotic leaks (OR, 2.787; 1.962–3.960) (Class II)^[76]^.

## Discussion

### Main findings

**Figure 5.**
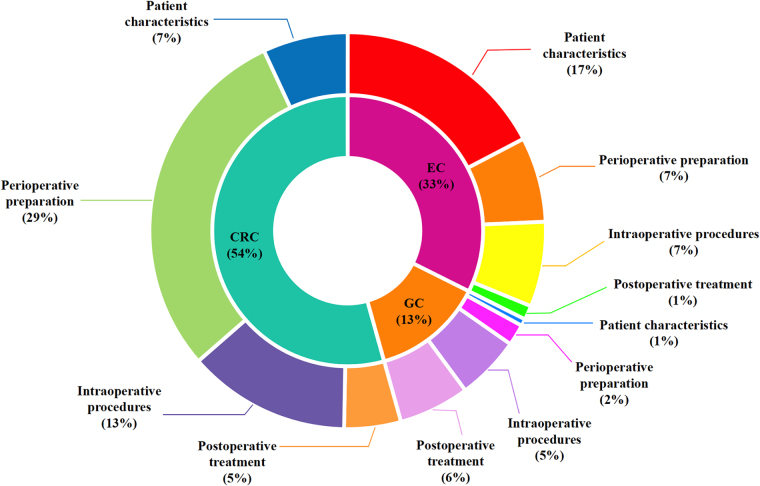
Map of distribution concerning outcomes of interest and various factors.

EC, esophageal cancer; GC, gastric cancer; CRC, colorectal cancer.

**Figure 6.**
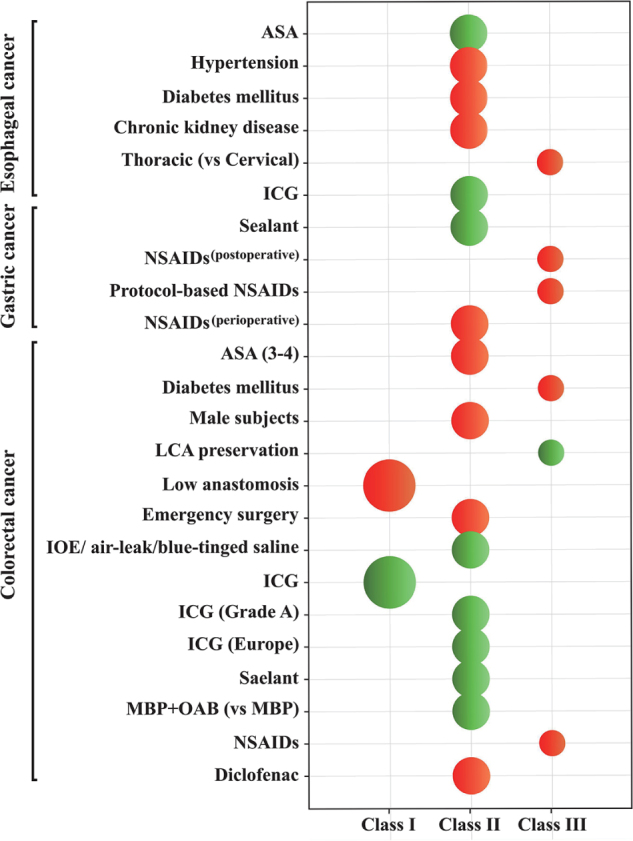
A schematic diagram of recommendations.

ASA, American Society of Anaesthesiologists; ICG, indocyanine green; NSAIDs, non-steroidal anti-inflammatory drugs; LCA, left colic artery; IOE, intraoperative endoscopy; MBP, mechanical bowel preparation; OAB, oral antibiotics;

To the best of our knowledge, this is the first umbrella review of observational studies and RCTs on protective and risk factors for postoperative anastomotic leakage in gastrointestinal surgery, based on a meta-analysis. A total of 173 factors were included in the study, covering patient characteristics, preoperative preparation, intraoperative procedures, and postoperative treatments. Among these factors, we identified 10 protective factors and 14 risk factors with high or moderate evidence levels (≥ III Class). The factors with evidence graded as Class IV included 35 risk factors and 14 protective factors, with relatively lower confidence in the effect size estimates. Additionally, there were 99 factors graded as Class V. The methodological quality of the studies included in the meta-analysis varied significantly (Fig. 5; Fig. 6).

**Colors:** Red indicates risk factors, green indicates protective factors; **Dot size**: Larger dots represent stronger levels of evidence.

#### Patient characteristics

In observational studies, ASA (3–4)^[4,5]^, male gender^[52]^, diabetes^[4]^, hypertension, and chronic kidney disease^[5]^ have been linked to an increased risk of postoperative anastomotic leakage. Furthermore, in this review, the relevance of factors has been supported by high-level evidence. Therefore, it is reasonable to conclude that ASA (3–4), male gender, diabetes, hypertension, and chronic kidney disease influence the risk of anastomotic leakage. Future similar studies are unlikely to alter this evidence. Patients with higher ASA scores are at a greater risk due to the increased incidence of comorbidities (such as cardiovascular or pulmonary diseases), which may impair tissue perfusion and oxygenation^[98]^. In male patients, the relatively narrow pelvic structure increases the difficulty of tissue dissection during surgery, potentially leading to postoperative complications. Additionally, differences in sex hormones are thought to affect intestinal microcirculation^[99]^, which may negatively impact anastomotic healing^[52,100,101]^. Moreover, age > 70 years, alcohol, chronic obstructive pulmonary disease (COPD), obesity, and cardiovascular diseases (ischemic heart disease, vascular diseases, and potential calcification of the aorta or mesenteric arteries)^[5,6,45,80]^ are supported by weak evidence.

#### Treatment-related factors

**Preoperative preparation**: Overall, the results of this umbrella review indicate that the use of oral antibiotics is the only factor in esophageal and gastrointestinal surgeries that is associated with a reduced risk of postoperative anastomotic leakage, reaching highly suggestive evidence^[63]^. Compared with mechanical bowel preparation alone, the combination of oral antibiotics can reduce potential pathogens in the intestinal contents, decrease microbial load at the anastomotic site, and lower the risk of postoperative infection. This is particularly important in mitigating the negative effects of bacterial toxins and inflammatory factors on anastomotic healing^[64,102]^.

**Intraoperative procedures**: Our study results indicate that surgical techniques such as low anterior resection of the rectum^[52]^, use of collagen or fibrin-based sealants^[16]^, ICG fluorescence imaging^[19]^, flexible endoscopic examination^[17,18]^, and leak tests using a Foley catheter and methylene blue test^[17,48]^ are significantly associated with a reduced risk of postoperative anastomotic leakage, reaching convincing or highly suggestive levels. ICG fluorescence imaging is an intraoperative real-time technique for assessing anastomotic blood supply. By intravenously injecting ICG and using near-infrared light to observe tissue perfusion, surgeons can optimize the anastomotic position and ensure adequate blood supply, thereby significantly reducing the risk of anastomotic leakage^[15,19]^. Furthermore, ICG can enhance the visibility of tumor lesions, improve lymph node detection rates, and reduce the incidence of anastomotic leakage^[103,104]^.

Our review highlights that collagen or fibrin-based sealants can significantly reduce the risk of postoperative anastomotic leakage. It has been shown that Faecalibacterium^[105]^ can interfere with collagen’s healing function by increasing collagenase activity, leading to anastomotic failure^[106,107]^. Microbial collagenase degrades the biological material framework, weakening the protective function of coatings. Sealants containing collagen fibrils and antimicrobial substances effectively prevent collagenase-induced damage and theoretically promote anastomotic healing and reduce leakage^[16]^.

Intraoperative flexible endoscopic examination, leak tests using a Foley catheter, and methylene blue testing are commonly used to identify potential anastomotic defects. Interestingly, our findings indicate that flexible endoscopy or Foley catheter testing alone is not significantly associated with reduced anastomotic leakage risk^[18,48]^. Although a negative result may lower the likelihood of postoperative leaks, it does not entirely exclude their occurrence. Compared to flexible endoscopy, Foley-based leak and methylene blue tests are simpler and more cost-effective. Flexible endoscopy, while offering direct visualization and greater accuracy in identifying leaks^[108,109]^, may cause mechanical trauma to the staple line during forced air insufflation. This can lead to false positives and potentially increase leakage risk^[17]^.

Our study results indicate that thoracic anastomosis is associated with a higher incidence of anastomotic leakage compared to cervical anastomosis, supported by suggestive evidence. However, many studies have reported a higher leakage rate with cervical anastomosis, albeit with fewer severe complications^[110,111]^. The higher leakage rate of cervical anastomosis may result from increased tension and limited blood supply at the gastric fundus, unlike thoracic anastomosis, which typically has lower tension and better vascularization^[112]^. Additionally, cervical anastomotic leaks are generally easier to manage and are associated with lower morbidity compared to thoracic leaks^[113,114]^.

In rectal cancer surgery, preserving the LCA significantly reduces the risk of postoperative anastomotic leakage, as suggested by our review. The blood supply to the anastomosis mainly comes from the marginal branches of the middle colic artery (MCA), and preserving the LCA improves blood flow to the proximal colon stump, lowering leakage risk^[49,115]^. Studies show that when the anastomosis is located less than 6 cm from the anal verge in low rectal cancer, leakage rates increase significantly^[52,116]^. Low rectal anastomosis often requires extensive mesenteric dissection to achieve a tension-free connection, which can impair local blood flow, increase mechanical stress, and hinder healing, raising the chance of leakage^[117]^. Compared with planned surgery, emergency resections due to comorbidities tend to involve more blood loss, transfusions, and use of vasoactive drugs, all of which further increase the risk of postoperative leakage^[4,99]^.

**Postoperative management:** Our results suggest a significant association between postoperative use of diclofenac^[76]^ and an increased risk of anastomotic leakage, with highly suggestive evidence. The use of NSAIDs, including regimen-based NSAIDs, also shows suggestive evidence linking them to an increased risk of anastomotic leakage^[21]^. Several studies have revealed the potential mechanisms through which NSAIDs may impair intestinal barrier function. Nonselective NSAIDs, particularly through the inhibition of cyclooxygenase (COX), especially COX-1, lead to mitochondrial dysfunction and increased epithelial permeability, which may facilitate bacterial invasion, trigger neutrophil infiltration, and promote excessive free-radical production^[118]^. Additionally, NSAIDs reduce the synthesis of protective prostaglandins, thereby weakening the intestinal immune barrier function^[119]^, and their acidic properties can directly damage the local mucosa^[120]^. Although selective COX-2 inhibitors may be better tolerated in normal gastrointestinal tissues, it has been suggested that both types of NSAIDs can weaken collagen fiber structure, decrease anastomotic tensile strength, and increase the risk of anastomotic rupture^[121,122]^. Furthermore, NSAIDs may inhibit epithelial cell migration and repair, further hindering intestinal healing^[123]^.

In inflammatory states, upregulation of COX-2 expression may promote healing by enhancing local blood supply through the production of prostaglandin E2 and vascular endothelial growth factor^[124]^. Our study shows that diclofenac significantly increases the incidence of anastomotic leakage following colorectal surgery^[76]^, while ketorolac shows no significant association^[77]^, suggesting that the impact of NSAIDs on anastomotic healing may be drug-specific. However, this finding contradicts the conclusion of a previous large-scale retrospective study^[23]^ that demonstrated no significant association between NSAID use and the incidence of anastomotic leakage following colorectal surgery. Furthermore, similar situations exist for intraoperative flexible endoscopic examination, leak testing through Foley catheters, and methylene blue testing^[17,18,45]^. Although our study incorporates relatively recent and comprehensive published data, these conflicting results underscore the necessity for more high-quality prospective studies to further elucidate these associations. However, it is undeniable that the use of nonsteroidal anti-inflammatory drugs should be cautious in clinical practice.

Moreover, it is noteworthy that current academic research intends to predominantly focus on treatment-related factors influencing gastrointestinal anastomotic leakage, while the impact of underlying diseases and lifestyle factors has received insufficient attention in clinical studies, resulting in their lower evidence levels in our analysis. However, these nontherapeutic factors are critically important, as they play a pivotal role in enhancing public health awareness, health education, and patient prognosis particularly among gastrointestinal cancer patients.

## Strengths and limitations

This study had several strengths. First, to the best of our knowledge, this is the first umbrella review to comprehensively analyze the factors associated with anastomotic leakage following gastrointestinal surgery. Second, compared with other studies summarizing gastrointestinal postoperative anastomotic leakage, this review employed strict standards for evidence evaluation, bias assessment, and methodological quality appraisal, providing a systematic and comprehensive evaluation.

In addition, this study had the following limitations. First, most of the included studies were observational, which means that the established associations may not necessarily indicate causation. While strong correlations were consistently identified across multiple studies, the possibility of confounding cannot be ruled out. Second, this study only included meta-analyses, potentially omitting factors not yet covered in the previous meta-analyses. Third, in key Item 2 of AMSTAR 2, some studies lacked preregistered protocols, affecting quality ratings. Fourth, only meta-analyses published in English were included, which may not represent the entirety of meta-analyses on postoperative anastomotic leakage. Fifth, some meta-analyses included a limited number of studies or had small sample sizes, which could have affected the statistical power and external validity of the findings. Sixth, most meta-analyses did not clearly distinguish between gastroenteric and enteric anastomotic leakage. To minimize the omission of factors related to postoperative anastomotic leakage, this study categorized postoperative anastomotic leakage by different surgical sites. Seventh, the meta-analyses included in this review exhibited significant variability in defining the location of anastomotic leakage. For example, some meta-analyses focused on anastomotic leakage following colorectal cancer surgery, others on gastric or esophageal cancer, while some did not differentiate and broadly categorized them under gastrointestinal diseases. What’s more, the primary outcomes of this study encompass anastomotic leakage following esophageal, gastrointestinal, and colorectal surgeries. However, it should be noted that diagnostic criteria and definitions of anastomotic leakage vary across these anatomical locations. Consequently, clinical application of our findings requires careful consideration of the specific surgical site involved. In addition, there exists no established consensus or protocol to guide the identification and management of literature with overlapping factors. This study referred methodologies from previously published high-quality studies to deal with potential overlaps^[38,40]^. We strongly advocate for the development of standardized guidelines to address this methodological challenge in future research. Additionally, the meta-analyses included in this review varied significantly in describing intervention strategies. For example, some broadly discussed NSAIDs, while others specifically examined diclofenac or ketorolac. Furthermore, this study systematically analyzed and synthesized findings from previous studies, revealing considerable heterogeneity in the available clinical evidence. Consequently, further high-quality clinical studies are warranted to establish more clinically meaningful and reliable conclusions.

## Summary and recommendations

This umbrella review indicates that the main factors associated with anastomotic leakage include 14 risk factors: ASA (3–4), male sex, diabetes, hypertension, chronic kidney disease, NSAIDs, nonselective NSAIDs (including diclofenac), low anastomoses (rectal cancer), thoracic anastomosis (esophageal cancer), and emergency surgery. In contrast, 10 protective factors were identified, including ICG (including grade A anastomotic leakage, European population), ASA (I or II), combined oral antibiotics and mechanical bowel preparation (vs. oral antibiotics alone), collagen or fibrin-based sealants, flexible endoscopic examination, leak tests via Foley catheter or methylene blue test, and preservation of the LCA. The evidence level for these postoperative anastomotic leakage-related factors is higher than for other factors in this study, making the results more reliable. Based on the above postoperative anastomotic leakage–related factors and the level of evidence, this study has compiled relevant recommendations for clinical practitioners and preventive experts (Table 1; Fig. 6). Further high-quality research is needed to confirm whether other factors in this study are closely associated with postoperative anastomotic leakage. In light of the above, both protective and risk factors related to postoperative anastomotic leakage require urgent attention, along with widespread public health education and guideline dissemination.

**Table 1:** Recommendations on preventing the risk of AL after esophageal, gastric, and colorectal surgery

Classification	Recommendations	Protective or harmful	Evidence class	Recommendation strength	Applicable surgical type
Patient characteristics	Control preoperative conditions such as hypertension, diabetes, and chronic kidney disease to reduce the risk of postoperative anastomotic leakage.	Risk	Class II	Highly suggestive evidence	Esophageal Colorectal
Preoperative preparation	For elective colorectal surgery, it is recommended to combine oral antibiotics with mechanical bowel preparation preoperatively to reduce the incidence of postoperative anastomotic leakage.	Protective	Class II	Highly suggestive evidence	Colorectal
	In esophageal cancer resection, preoperative gastric ischemic preconditioning for more than 2 weeks can significantly reduce the risk of postoperative anastomotic leakage.	Protective	Class II	Highly suggestive evidence	Esophageal
	Neoadjuvant therapy may increase the risk of anastomotic leakage after gastrointestinal surgery. Therefore, when selecting a neoadjuvant therapy regimen, it is essential to comprehensively evaluate the patient’s tumor characteristics and overall condition, further optimize anastomotic techniques during surgery, and enhance postoperative monitoring to reduce the incidence of anastomotic leakage.	Risk	Class III	Suggestive evidence	Gastric
Intraoperative procedures	It is recommended to use indocyanine green fluorescence imaging during surgery to assess vascular perfusion at the anastomotic site, which can reduce the incidence of postoperative anastomotic leakage.	Protective	Class I	Strongly suggestive	Esophageal Colorectal
	The application of reinforcement materials, such as collagen or fibrin-based sealants, at the anastomotic site to strengthen the anastomosis can reduce the incidence of postoperative AL.	Protective	Class II	Highly suggestive evidence	Gastric Colorectal
	The combined use of flexible endoscopy, leak tests with Foley catheter, and methylene blue tests can reduce the incidence of postoperative anastomotic leakage; however, their effectiveness is limited when used individually.	Protective	Class II	Highly suggestive evidence	Colorectal
	It is recommended to preserve the left colic artery during surgery to improve the blood supply to the colorectal anastomosis and reduce the incidence of postoperative anastomotic leakage. However, the decision should be carefully balanced based on the patient’s specific condition.	Protective	Class III	Suggestive evidence	Low rectal
	In patients with esophageal cancer, the incidence of anastomotic leakage is significantly higher in those undergoing transthoracic anastomosis than in those undergoing cervical anastomosis, and careful consideration should be given to the choice of surgical approach.	Risk	Class III	Suggestive evidence	Esophageal
	For patients with low rectal cancer, the risk of anastomotic leakage significantly increases postoperatively.	Risk	Class III	Suggestive evidence	Low rectal
	The risk of anastomotic leakage in emergency surgery patients is significantly higher than that in elective surgery patients. Therefore, for emergency surgery patients, meticulous intraoperative techniques should be emphasized to ensure the quality of the anastomosis, and close postoperative monitoring is necessary to promptly identify and manage potential complications.	Risk	Class III	Suggestive evidence	Colorectal
Perioperative treatment	Minimize the use of NSAIDs during the perioperative period, as they may increase the risk of AL.	Risk	Class II	Highly suggestive evidence	Gastric Colorectal

## Supplementary Material

**Figure:**
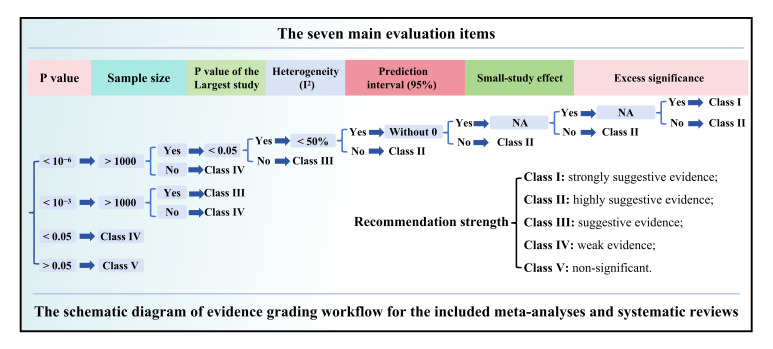


## Data Availability

The data are publicly available and there are no restrictions. Data sources: Four databases including PubMed, Embase, Cochrane Library, and Web of Science databases up to November 2024. Data sharing: The data from this study can be shared with other researchers. Data processing and analysis: Literature screening, quality evaluation, and data extraction were performed according to inclusion and exclusion criteria. The data were analyzed using StataMP 18 and GraphPad Prism 10. Data protection and privacy: The data in this study comply with applicable legal, ethical and privacy regulations. Repeatability of data: The data in this study are reproducible and robust.

## Research registration unique identifying number (UIN)

This study has been registered at PROSPERO. Registration ID: CRD42024628648. (https://www.crd.york.ac.uk/PROSPERO).
