# Supplementary material for: Risk and protective factors for postoperative anastomotic leakage in esophageal and gastrointestinal surgery: an umbrella review of meta-analyses and systematic reviews
Source: Int J Surg. 2025 Sep 19;112(1):1722–36. doi: 10.1097/JS9.0000000000003308 (PMC12825836; doi:10.1097/JS9.0000000000003308)
Supplement: Supplementary file 1 [file js9-112-1722-001.docx]

| **Table S1. Evidence classification criteria.** | |
| --- | --- |
| **Evidence class** | Description |
| Class I:  strongly suggestive evidence | >1000 cases (or >20 000 participants for continuous outcomes); statistical significance at P <10 −6 (random effects); no evidence of small study effects and excess significance bias; 95% prediction interval excluded null value; no large heterogeneity (I ^2^ <50%) |
| Class II:  highly suggestive evidence | >1000 cases (or >20 000 participants for continuous outcomes), statistical significance at P <10 −6 (random effects), and largest study with 95% confidence interval excluding |
| Class III:  suggestive evidence | >1000 cases (or >20 000 participants for continuous outcomes) and statistical significance at P <0.001 |
| Class IV:  weak evidence | Only significant associations with P <0.05 |
| Class V:  non-significant | P >0.05 |
