# Supplementary material for: Risk and protective factors for postoperative anastomotic leakage in esophageal and gastrointestinal surgery: an umbrella review of meta-analyses and systematic reviews
Source: Int J Surg. 2025 Sep 19;112(1):1722–36. doi: 10.1097/JS9.0000000000003308 (PMC12825836; doi:10.1097/JS9.0000000000003308)
Supplement: Supplementary file 2 [file js9-112-1722-003.docx]

|  | **ITEMS**  **Table S5. Quality assessment of included meta-analyses with AMSTAR 2.** | | | | | | | | | | | |  |  |  |  | **Total**  **AMSTAR**  **Score** | **Final rating** |
| --- | --- | --- | --- | --- | --- | --- | --- | --- | --- | --- | --- | --- | --- | --- | --- | --- | --- | --- |
|  | **1** | **2** | **3** | **4** | **5** | **6** | **7** | **8** | **9** | **10** | **11** | **12** | **13** | **14** | **15** | **16** |  |  |
| (Wu et al. 2013)^80^ | Y | pY | N | pY | Y | Y | N | pY | pY | N | Y | Y | Y | Y | Y | Y | 13 | Low |
| *(Mao et al. 2020)^86^* | Y | pY | N | pY | Y | Y | N | pY | pY | N | Y | Y | Y | Y | Y | Y | 13 | Low |
| (Zong et al. 2011)^87^ | Y | pY | N | pY | Y | Y | N | pY | pY | N | Y | Y | Y | Y | Y | Y | 13 | Low |
| (Syn et al. 2019)^88^ | Y | pY | N | pY | Y | Y | N | pY | pY | N | Y | Y | Y | Y | Y | Y | 13 | Low |
| (Wang et al. 2019)^91^ | Y | pY | N | pY | Y | Y | N | pY | pY | N | Y | Y | Y | Y | Y | Y | 13 | Low |
| (Zhao et al. 2021)^56^ | Y | pY | N | pY | Y | Y | N | pY | Y | N | Y | Y | Y | Y | Y | Y | 13 | Low |
| (Cira et al. 2022)^16^ | Y | pY | N | pY | Y | Y | N | pY | pY | N | Y | Y | Y | Y | Y | Y | 13 | Low |
| (Pang et al. 2023)^93^ | Y | pY | N | pY | Y | Y | N | pY | pY | N | Y | Y | Y | Y | Y | Y | 13 | Low |
| (Liu et al. 2022)^73^ | Y | N | N | pY | Y | Y | N | pY | N | N | Y | N | N | Y | Y | Y | 9 | Very Low |
| (Jamjittrong et al. 2020)^21^ | Y | N | N | pY | Y | Y | N | pY | pY | N | Y | N | N | Y | Y | Y | 10 | Very Low |
| (Wang et al. 2015)^94^ | Y | pY | N | pY | Y | Y | N | pY | pY | N | Y | N | N | Y | Y | Y | 11 | Very Low |
| (He et al. 2022)^20^ | Y | pY | Y | pY | Y | Y | N | pY | pY | N | Y | Y | Y | Y | Y | Y | 14 | Low |
| (Osland et al. 2011)^79^ | Y | pY | Y | pY | Y | Y | N | pY | pY | N | Y | Y | Y | Y | Y | Y | 14 | Low |
| (Yu et al. 2021)^22^ | N | pY | N | pY | Y | Y | N | N | pY | N | Y | Y | Y | Y | Y | Y | 11 | Low |
| (Peng et al. 2016)^75^ | Y | pY | N | pY | Y | Y | N | pY | pY | N | Y | Y | Y | Y | Y | Y | 13 | Low |
| (Kamarajah et al. 2020)^5^ | Y | pY | N | pY | Y | Y | N | pY | pY | N | Y | N | N | Y | N | Y | 10 | Very Low |
| (Mengardo et al. 2018)^81^ | Y | pY | N | N | N | N | N | pY | pY | N | Y | N | N | Y | N | Y | 7 | Very Low |
| (Hoek et al. 2020)^6^ | Y | pY | N | pY | Y | Y | N | pY | pY | N | Y | Y | Y | Y | Y | Y | 13 | Low |
| (Schizas et al. 2020)^82^ | Y | pY | N | pY | Y | Y | N | pY | pY | N | Y | Y | Y | Y | Y | Y | 13 | Low |
| (Michalinos et al. 2020)^11^ | Y | pY | N | pY | Y | Y | N | pY | pY | N | Y | Y | Y | Y | Y | Y | 13 | Low |
| (Zhuo et al. 2020)^12^ | Y | pY | N | pY | Y | Y | N | pY | pY | N | Y | Y | Y | Y | Y | Y | 13 | Low |
| (Kumagai et al. 2014)^83^ | Y | pY | N | pY | N | N | N | pY | pY | N | Y | Y | Y | Y | Y | Y | 11 | Low |
| (Qin et al. 2018)^84^ | Y | pY | N | pY | Y | Y | N | pY | pY | N | Y | Y | Y | Y | Y | Y | 13 | Low |
| (Lin et al. 2017)^85^ | Y | pY | N | pY | Y | Y | N | pY | pY | N | Y | N | N | Y | N | Y | 10 | Very Low |
| (Scheufele et al. 2020)^13^ | Y | pY | N | pY | Y | Y | N | pY | pY | N | Y | Y | Y | Y | Y | Y | 13 | Low |
| (Wang et al. 2018)^89^ | Y | pY | N | pY | Y | Y | N | pY | pY | N | Y | Y | Y | Y | Y | Y | 13 | Low |
| (Shen et al. 2021)^90^ | Y | pY | N | pY | Y | Y | N | pY | pY | N | Y | Y | Y | Y | Y | Y | 13 | Low |
| (Nienhüser et al. 2022)^92^ | Y | pY | N | pY | Y | Y | N | pY | pY | N | Y | Y | Y | Y | Y | Y | 13 | Low |
| (Casas et al. 2022)^15^ | Y | pY | N | pY | Y | Y | N | pY | N | N | Y | N | N | Y | N | N | 8 | Very Low |
| (Grigor et al. 2021)^14^ | Y | pY | Y | pY | Y | Y | Y | pY | pY | N | Y | Y | Y | Y | Y | Y | 15 | High |
| (Weijs et al. 2017)^95^ | Y | pY | N | pY | Y | Y | N | pY | pY | N | Y | Y | Y | Y | Y | Y | 13 | Low |
| (Abdelrahman et al. 2022)^96^ | Y | pY | N | pY | Y | Y | N | pY | pY | N | Y | N | N | Y | N | Y | 10 | Very Low |
| (Dias et al. 2022)^4^ | Y | pY | N | pY | Y | Y | N | pY | pY | N | Y | Y | Y | Y | Y | Y | 13 | Low |
| (Nugent et al. 2021)^45^ | Y | pY | N | pY | Y | Y | N | pY | pY | N | Y | Y | Y | Y | Y | Y | 13 | Low |
| (Trejo-Avila et al. 2021)^46^ | Y | pY | N | pY | Y | Y | N | pY | pY | N | Y | Y | Y | Y | Y | Y | 13 | Low |
| (Pommergaard et al. 2014)^52^ | Y | pY | N | pY | N | N | N | pY | pY | N | Y | Y | Y | Y | Y | Y | 11 | Low |
| (Yang et al. 2015)^47^ | Y | pY | N | pY | Y | Y | N | pY | Y | N | Y | Y | Y | Y | Y | Y | 13 | Low |
| (Wu et al. 2016)^48^ | Y | pY | N | pY | Y | Y | N | pY | Y | N | Y | Y | Y | Y | Y | Y | 13 | Low |
| (Fan et al. 2018)^49^ | Y | pY | Y | pY | Y | Y | N | pY | pY | N | Y | Y | Y | Y | Y | Y | 14 | Low |
| (Cirocchi et al. 2012)^50^ | Y | pY | N | pY | Y | Y | N | pY | Y | N | Y | Y | Y | Y | Y | Y | 13 | Low |
| (Zeng and Su 2018)^51^ | Y | pY | N | pY | Y | Y | N | pY | pY | N | Y | Y | Y | Y | Y | Y | 13 | Low |
| (Wang et al. 2020)^7^ | Y | pY | N | pY | Y | Y | N | pY | pY | N | Y | Y | Y | Y | Y | Y | 13 | Low |
| (Ahmad et al. 2021)^53^ | Y | pY | N | pY | Y | Y | N | pY | pY | N | Y | Y | Y | Y | Y | Y | 13 | Low |
| (Xia, Wu, Ma, et al. 2023)^9^ | Y | pY | N | pY | Y | Y | N | pY | pY | N | Y | Y | Y | Y | Y | Y | 13 | Low |
| (Zhang et al. 2016)^54^ | Y | pY | N | pY | Y | Y | N | pY | pY | N | Y | Y | Y | Y | Y | Y | 13 | Low |
| (Rondelli et al. 2021)^8^ | Y | pY | N | pY | Y | Y | N | pY | pY | N | Y | N | N | Y | N | Y | 10 | Very Low |
| (Fahy et al. 2021)^97^ | Y | pY | N | pY | Y | Y | N | pY | pY | N | Y | N | N | Y | N | N | 9 | Very Low |
| (Liu et al. 2021)^55^ | Y | pY | N | pY | Y | Y | N | pY | pY | N | Y | Y | Y | Y | Y | Y | 13 | Low |
| (Kryzauskas et al. 2020)^17^ | Y | pY | N | pY | Y | Y | N | pY | pY | N | Y | Y | Y | Y | Y | Y | 13 | Low |
| (Aly et al. 2019)^18^ | Y | pY | N | pY | Y | Y | N | pY | pY | N | Y | Y | Y | Y | Y | Y | 13 | Low |
| (Tang et al. 2021)^10^ | Y | pY | N | pY | Y | Y | N | pY | pY | N | Y | N | N | Y | N | Y | 10 | Very Low |
| (Xia, Wu, Luo, et al. 2023)^19^ | Y | pY | N | pY | Y | Y | N | pY | pY | N | Y | Y | Y | Y | Y | Y | 13 | Low |
| (Wiggins et al. 2015)^57^ | Y | pY | N | pY | N | N | N | pY | pY | N | Y | N | N | Y | N | Y | 8 | Very Low |
| (Geng et al. 2015)^58^ | Y | pY | N | pY | Y | Y | N | pY | pY | N | Y | Y | Y | Y | Y | N | 12 | Low |
| (Archampong, Borowski, and Dickinson 2010)^59^ | N | pY | N | pY | Y | Y | N | pY | pY | N | Y | Y | Y | Y | Y | Y | 12 | Low |
| (Dahabreh et al. 2015)^60^ | Y | pY | N | pY | Y | Y | N | pY | pY | N | Y | Y | Y | Y | Y | Y | 13 | Low |
| (Qin et al. 2014)^61^ | Y | pY | N | pY | Y | Y | N | pY | pY | N | Y | Y | Y | Y | Y | Y | 13 | Low |
| (Rollins, Javanmard-Emamghissi, and Lobo 2018)^62^ | Y | pY | N | pY | Y | Y | N | pY | pY | N | Y | Y | Y | Y | Y | Y | 13 | Low |
| (Leenen, Hentzen, and Ockhuijsen 2019)^63^ | Y | pY | N | pY | Y | Y | N | pY | pY | N | Y | Y | Y | Y | Y | Y | 13 | Low |
| (Rollins et al. 2019)^64^ | Y | pY | N | pY | Y | Y | N | pY | pY | N | Y | Y | Y | Y | Y | N | 12 | Low |
| (Bellows et al. 2011)^65^ | Y | pY | N | pY | Y | Y | N | pY | pY | N | Y | Y | Y | Y | Y | N | 12 | Low |
| (Johnson et al. 2023)^66^ | Y | pY | N | pY | Y | Y | N | pY | pY | N | Y | Y | Y | Y | Y | Y | 13 | Low |
| (Srinivasa et al. 2011)^67^ | Y | pY | N | pY | Y | Y | N | pY | pY | N | Y | N | N | Y | N | Y | 10 | Very Low |
| (Hu et al. 2017)^68^ | Y | pY | N | pY | Y | Y | N | pY | pY | N | Y | Y | Y | Y | Y | N | 12 | Low |
| (Du et al. 2018)^69^ | Y | pY | N | pY | Y | Y | N | pY | pY | N | Y | Y | Y | Y | Y | Y | 13 | Low |
| (Yang et al. 2022)^70^ | Y | pY | N | pY | Y | Y | N | pY | pY | N | Y | Y | Y | Y | Y | Y | 13 | Low |
| (Wang et al. 2017)^71^ | Y | pY | N | pY | Y | Y | N | pY | pY | N | Y | Y | Y | Y | Y | Y | 13 | Low |
| (Sangiorgio et al. 2022)^72^ | Y | pY | N | pY | Y | Y | N | pY | pY | N | Y | Y | Y | Y | Y | Y | 13 | Low |
| (Yang, Li, and Du 2021)^74^ | Y | pY | N | pY | Y | Y | N | pY | pY | N | Y | Y | Y | Y | Y | Y | 13 | Low |
| (Modasi et al. 2019)^76^ | Y | pY | N | pY | Y | Y | N | pY | pY | N | Y | Y | Y | Y | Y | N | 12 | Low |
| (Kastora et al. 2021)^77^ | Y | pY | N | pY | Y | Y | N | pY | pY | N | Y | Y | Y | Y | Y | Y | 13 | Low |
| (Ouyang et al. 2019)^78^ | Y | pY | N | pY | Y | Y | Y | pY | pY | N | Y | Y | Y | Y | Y | Y | 14 | Low |

**AMSTAR 2 items assessed (items in italic are considered critical):**

1, PICO description; 2, protocol registered before the commencement of the review; 3, study design included in the review; 4, adequacy of the literature search; 5, two authors study selection; 6, two authors study extraction; 7, justification for excluding individual studies; 8, included studies descripted in detail; 9, risk of bias for the single studies being included in the review; 10, source of funding of primary studies; 11, appropriateness of meta-analytical methods; 12, impact of risk of bias of single studies on the results of the meta-analysis; 13, consideration of risk of bias when interpreting the results of the review; 14 explanation and discussion of the heterogeneity observed; 15, assessment of presence and likely impact of publication bias; 16, funding sources and conflict of interest declared.

**Abbreviations:** Y, yes; pY, partial yes; N, no.

**Footnotes:**

**High:** 0–1 non-critical weakness. The systematic review provides an accurate and comprehensive summary of the results of the available studies that address the question of interest.

**Moderate:** >1 non-critical weakness. The systematic review has more than one weakness, but no critical flaws. It may provide an accurate summary of the results of the available studies that were included in the review.

**Low:** 1 critical flaw with or without non-critical weaknesses. The review has a critical flaw and may not provide an accurate and comprehensive summary of the available studies that address the question of interest.

**Critically low:** >1 critical flaw with or without non-critical weaknesses. The review has more than one critical flaw and should not be relied on to provide an accurate and comprehensive summary of the available studies.
