# Supplementary material for: Risk and protective factors for postoperative anastomotic leakage in esophageal and gastrointestinal surgery: an umbrella review of meta-analyses and systematic reviews
Source: Int J Surg. 2025 Sep 19;112(1):1722–36. doi: 10.1097/JS9.0000000000003308 (PMC12825836; doi:10.1097/JS9.0000000000003308)
Supplement: Supplementary file 3 [file js9-112-1722-004.docx]

| **Factors**  **Table S2. Characteristics and quality assessment of the meta-analyses investigating protective and dangerous factors concerning Esophageal carcinoma. Associations reported in italic are those retained in the main analysis. Furthermore, significant associations(P<0.05) are presented in bold.** | **Patients** | E**vents or cases/total(n.)** | **Studies(n.)** | | | **Summary odds ratio estimate(OR,95% CI)** | | | **Selected effect model** | **Tau^2^** | **P value** | | | **95% PI** | **Heterogeneity** | | **Egger's**  **P value** | **Excess**  **significance** | **Small-study effect** | **Evidence class** | **AMSTAR 2** |
| --- | --- | --- | --- | --- | --- | --- | --- | --- | --- | --- | --- | --- | --- | --- | --- | --- | --- | --- | --- | --- | --- |
|  |  |  | **Total** | **RCT** | **OE** | **Fixed effects** | **Random effects** | **Largest study** |  |  | **Fixed** | **Random** | **Largest** |  | **I^2^(%)** | **I^2^ -P value** |  |  |  |  |  |
| **Various factors vs. placebo or conventional treatment** | | |  |  |  |  |  |  |  |  |  |  |  |  |  |  |  |  |  |  |  |
| **Patient characteristics** | |  |  |  |  |  |  |  |  |  |  |  |  |  |  |  |  |  |  |  |  |
| ASA (I/II)^5^ | esophageal cancer | 2421/19721 | 12 | 0 | 12 | 0.658(0.594 to 0.729) | ***0.501(0.380 to 0.660)*** | 0.169(0.099 to 0.290) | Random | 0.1474 | 0.000 | 0.000 | 0.000 | 0.718 to 7.243 | 79.9 | 0.000 | 0.063 | No | Yes | II | Very Low |
| Age>70^5^ | esophageal cancer | 924/9002 | 17 | 0 | 17 | ***1.193(1.001 to 1.421)*** | 1.194(1.002 to 1.424) | 1.105(0.829 to 1.473) | Fixed | 0.0000 | 0.048 | 0.048 | 0.495 | 1.592 to 3.266 | 0.0 | 1.000 | 0.171 | No | No | IV | Very Low |
| BMI<30^81^ | esophageal cancer | 305/2560 | 7 | 0 | 7 | ***0.622(0.464 to 0.833)*** | 0.614(0.438 to 0.860) | 0.111(0.020 to 0.598) | Fixed | 0.0419 | 0.001 | 0.005 | 0.011 | 0.257 to 20.211 | 20.3 | 0.275 | 0.390 | Yes | No | IV | Very Low |
| BMI>30^5^ | esophageal cancer | 1304/18738 | 15 | 0 | 15 | 0.321(0.280 to 0.368) | *1.111(0.454 to 2.722)* | 0.018(0.004 to 0.078) | Random | 2.7040 | 0.000 | 0.818 | 0.000 | 0.027 to 192.500 | 95.8 | 0.000 | 0.017 | Yes | Yes | V | Very Low |
| Alcohol history^5^ | esophageal cancer | 333/3238 | 3 | 0 | 3 | ***1.371(1.071 to 1.755)*** | 1.360(1.062 to 1.742) | 1.294(0.943 to 1.776) | Fixed | 0.0000 | 0.012 | 0.015 | 0.111 | 1.535 to 3.387 | 0.0 | 0.588 | 0.030 | No | Yes | IV | Very Low |
| COPD^5^ | esophageal cancer | 1004/5979 | 7 | 0 | 7 | ***1.498(1.235 to 1.817)*** | 1.586(1.106 to 2.273) | 1.466(1.165 to 1.846) | Fixed | 0.0780 | 0.000 | 0.012 | 0.001 | 1.085 to 4.790 | 38.2 | 0.138 | 0.412 | Yes | Yes | IV | Very Low |
| FEV1 (% predicted)^5^ | esophageal cancer | NR | 4 | 0 | 4 | ***-4.489(-7.069 to -1.909)*** | -4.489(-7.069 to -1.909) | -4.000(-7.329 to -0.671) | Fixed | 0.0000 | 0.001 | 0.001 | 0.019 | NA | 0.0 | 0.888 | 0.962 | Yes | No | IV | Very Low |
| Hypertension^5^ | esophageal cancer | 1951/15637 | 9 | 0 | 9 | 1.386(1.253 to 1.534) | ***1.467(1.192 to 1.806)*** | 1.238(1.035 to 1.480) | Random | 0.0403 | 0.000 | 0.000 | 0.019 | 1.328 to 3.913 | 56.7 | 0.018 | 0.341 | No | No | II | Very Low |
| Ischaemic heart disease^5^ | esophageal cancer | 1194/11263 | 7 | 0 | 7 | ***1.244(1.056 to 1.465)*** | 1.257(1.067 to 1.482) | 1.318(1.105 to 1.573) | Fixed | 0.0000 | 0.009 | 0.006 | 0.002 | 1.828 to 2.843 | 0.0 | 0.577 | 0.116 | No | Yes | IV | Very Low |
| Cardiac arrhythmia^5^ | esophageal cancer | 789/4249 | 3 | 0 | 3 | 1.574(1.234 to 2.008) | ***1.824(1.030 to 3.230)*** | 1.567(1.183 to 2.076) | Random | 0.1550 | 0.000 | 0.039 | 0.002 | 0.817 to 6.365 | 65.7 | 0.054 | 0.498 | No | Yes | IV | Very Low |
| Any cardiac diseases^5^ | esophageal cancer | 1100/9582 | 8 | 0 | 8 | ***1.527(1.220 to 1.911)*** | 1.461(1.048 to 2.037) | 1.655(1.120 to 2.446) | Fixed | 0.0648 | 0.000 | 0.025 | 0.011 | 1.033 to 5.034 | 34.0 | 0.157 | 0.031 | No | Yes | IV | Very Low |
| Vascular disease^5^ | esophageal cancer | 1595/11347 | 5 | 0 | 5 | 1.622(1.308 to 2.011) | ***1.768(1.184 to 2.639)*** | 1.657(1.245 to 2.205) | Random | 0.1006 | 0.000 | 0.005 | 0.001 | 0.969 to 5.363 | 54.8 | 0.065 | 0.592 | No | Yes | IV | Very Low |
| Diabetes mellitus^5^ | esophageal cancer | 1528/10513 | 15 | 0 | 15 | ***1.639(1.407 to 1.909)*** | 1.670(1.433 to 1.946) | 1.487(1.194 to 1.851) | Fixed | 0.0000 | 0.000 | 0.000 | 0.000 | 1.734 to 2.999 | 0.0 | 0.548 | 0.102 | No | Yes | II | Very Low |
| Chronic kidney disease^5^ | esophageal cancer | 912/8524 | 4 | 0 | 4 | ***3.103(2.141 to 4.497)*** | 3.126(2.157 to 4.532) | 2.932(1.949 to 4.410) | Fixed | 0.0000 | 0.000 | 0.000 | 0.000 | 1.369 to 3.798 | 0.0 | 0.846 | 0.066 | No | Yes | II | Very Low |
| Non Sarcopenia^82^ | esophageal cancer | 176/2128 | 5 | 0 | 5 | 0.742(0.543 to 1.012) | *0.753(0.421 to 1.346)* | 0.184(0.062 to 0.547) | Random | 0.2687 | 0.060 | 0.339 | 0.002 | 0.354 to 14.666 | 64.2 | 0.025 | 0.783 | Yes | Yes | V | Low |
| Use Steroid^5^ | esophageal cancer | 939/8195 | 4 | 0 | 4 | 2.360(1.591 to 3.503) | ***5.089(1.392 to 18.611)*** | 1.744(1.094 to 2.781) | Random | 1.1853 | 0.000 | 0.014 | 0.019 | 0.149 to 34.971 | 75.2 | 0.007 | 0.152 | Yes | No | IV | Very Low |
| THAO calcifification^6^ | esophageal cancer | 342/1820 | 5 | 0 | 5 | 2.143(1.657 to 2.771) | ***2.182(1.290 to 3.692)*** | 1.098(0.635 to 1.900) | Random | 0.2358 | 0.000 | 0.004 | 0.738 | 0.578 to 8.998 | 71.2 | 0.008 | 0.919 | Yes | No | IV | Low |
| CA calcification^6^ | esophageal cancer | 342/1821 | 5 | 0 | 5 | 1.646(1.272 to 2.131) | ***1.571(1.023 to 2.410)*** | 1.505(0.962 to 2.355) | Random | 0.1335 | 0.000 | 0.039 | 0.073 | 0.793 to 6.552 | 58.9 | 0.045 | 0.343 | No | No | IV | Low |
| RPCA calcification^6^ | esophageal cancer | 296/1661 | 5 | 0 | 5 | ***2.626(1.291 to 5.342)*** | 2.692(1.268 to 5.717) | 1.189(0.251 to 5.632) | Fixed | 0.0000 | 0.008 | 0.010 | 0.828 | 0.326 to 15.933 | 0.0 | 0.572 | 0.996 | Yes | No | IV | Low |
| LPCA calcification^6^ | esophageal cancer | 296/1661 | 5 | 0 | 5 | *1.282(0.883 to 1.863)* | 1.329(0.816 to 2.165) | 0.898(0.444 to 1.813) | Fixed | 0.0948 | 0.191 | 0.253 | 0.763 | 0.716 to 7.263 | 31.5 | 0.211 | 0.148 | Yes | No | V | Low |
| THAO calcium score 2 (vs 0)^6^ | esophageal cancer | 134/710 | 4 | 0 | 4 | ***2.185(1.477 to 3.233)*** | 2.256(1.249 to 4.076) | 1.278(0.567 to 2.881) | Fixed | 0.1619 | 0.000 | 0.007 | 0.553 | 0.554 to 9.391 | 45.3 | 0.139 | 0.688 | No | No | IV | Low |
| CA calcium score 2 (vs 0)^6^ | esophageal cancer | 162/863 | 4 | 0 | 4 | *1.500(0.950 to 2.368)* | 1.527(0.962 to 2.422) | 1.578(0.862 to 2.887) | Fixed | 0.0000 | 0.082 | 0.072 | 0.139 | 1.071 to 4.853 | 0.0 | 0.844 | 0.138 | No | No | V | Low |
| THAO calcification present (cervical)^6^ | esophageal cancer | 223/1079 | 2 | 0 | 2 | 2.385(1.730 to 3.287) | ***2.352(1.268 to 4.363)*** | 1.693(1.036 to 2.767) | Random | 0.1455 | 0.000 | 0.007 | 0.036 | 0.745 to 6.975 | 73.0 | 0.054 | NA | No | NA | IV | Low |
| CA calcification present (cervical)^6^ | esophageal cancer | 223/1079 | 2 | 0 | 2 | 2.050(1.478 to 2.845) | ***2.145(1.061 to 4.339)*** | 1.505(0.962 to 2.355) | Random | 0.2035 | 0.000 | 0.034 | 0.073 | 0.661 to 7.869 | 78.8 | 0.030 | NA | Yes | NA | IV | Low |
| RPCA calcification (cervical)^6^ | esophageal cancer | 177/919 | 2 | 0 | 2 | ***4.962(1.676 to 14.687)*** | 4.985(1.671 to 14.866) | 4.223(1.239 to 14.392) | Fixed | 0.0000 | 0.004 | 0.004 | 0.021 | 0.492 to 10.558 | 0.0 | 0.559 | NA | No | NA | IV | Low |
| LPCA calcification (cervical)^6^ | esophageal cancer | 177/919 | 2 | 0 | 2 | 1.583(0.852 to 2.943) | *3.323(0.330 to 33.453)* | 1.285(0.658 to 2.508) | Random | 2.1757 | 0.146 | 0.308 | 0.463 | 0.056 to 93.083 | 74.9 | 0.046 | NA | Yes | NA | V | Low |
| THAO calcification (thoracic)^6^ | esophageal cancer | 54/331 | 2 | 0 | 2 | ***3.728(1.800 to 7.720)*** | 3.401(1.205 to 9.601) | 1.860(0.558 to 6.201) | Fixed | 0.2682 | 0.000 | 0.021 | 0.312 | 0.318 to 16.325 | 47.0 | 0.170 | NA | No | NA | IV | Low |
| CA calcifcation (thoracic)^6^ | esophageal cancer | 54/331 | 2 | 0 | 2 | *1.103(0.605 to 2.011)* | 1.103(0.605 to 2.011) | 1.106(0.541 to 2.262) | Fixed | 0.0000 | 0.750 | 0.750 | 0.782 | 0.932 to 5.575 | 0.0 | 0.987 | NA | No | NA | V | Low |
| RPCA calcifification (thoracic)^6^ | esophageal cancer | 54/331 | 2 | 0 | 2 | *1.905(0.477 to 7.606)* | 1.901(0.470 to 7.689) | 2.175(0.350 to 13.504) | Fixed | 0.0000 | 0.362 | 0.367 | 0.404 | 0.233 to 22.358 | 0.0 | 0.822 | NA | Yes | NA | V | Low |
| LPCA calcification (thoracic)^6^ | esophageal cancer | 54/331 | 2 | 0 | 2 | *1.412(0.743 to 2.685)* | 1.420(0.745 to 2.705) | 1.644(0.753 to 3.587) | Fixed | 0.0000 | 0.292 | 0.287 | 0.212 | 0.859 to 6.048 | 0.0 | 0.514 | NA | No | NA | V | Low |
| **Preoperative preparation** | |  |  |  |  |  |  |  |  |  |  |  |  |  |  |  |  |  |  |  |  |
| Ischemic preconditioning^11^ | esophageal cancer | 223/1612 | 15 | 0 | 15 | *0.731(0.533 to 1.004)* | 0.730(0.521 to 1.023) | 0.175(0.044 to 0.701) | Fixed | 0.0148 | 0.053 | 0.068 | 0.014 | 0.394 to 13.195 | 3.2 | 0.416 | 0.376 | Yes | Yes | V | Low |
| LGA only or SGA only^11^ | esophageal cancer | 64/432 | 4 | 0 | 4 | *0.740(0.424 to 1.293)* | 0.754(0.407 to 1.399) | 0.418(0.146 to 1.194) | Fixed | 0.0372 | 0.291 | 0.371 | 0.103 | 0.565 to 9.207 | 8.8 | 0.349 | 0.431 | Yes | Yes | V | Low |
| Laparoscopic ligation^12^ | esophageal cancer | 180/1473 | 10 | 0 | 10 | ***0.662(0.451 to 0.972)*** | 0.718(0.482 to 1.069) | 0.463(0.175 to 1.226) | Fixed | 0.0000 | 0.035 | 0.103 | 0.121 | 0.618 to 8.410 | 0.0 | 0.532 | 0.355 | No | No | V | Low |
| Embolization^11^ | esophageal cancer | 74/414 | 6 | 0 | 6 | *0.785(0.439 to 1.404)* | 0.585(0.217 to 1.582) | 0.175(0.044 to 0.701) | Fixed | 0.6396 | 0.415 | 0.291 | 0.014 | 0.167 to 31.129 | 46.3 | 0.097 | 0.417 | Yes | Yes | V | Low |
| LGA+SA (embolization) or SGA (ligation)^11^ | esophageal cancer | 159/1189 | 12 | 0 | 12 | *0.739(0.504 to 1.083)* | 0.720(0.468 to 1.108) | 0.175(0.044 to 0.701) | Fixed | 0.0434 | 0.121 | 0.135 | 0.014 | 0.375 to 18.487 | 7.3 | 0.374 | 0.353 | Yes | Yes | V | Low |
| IC (<1w)^12^ | esophageal cancer | 36/441 | 2 | 0 | 2 | *0.732(0.374 to 1.435)* | 0.741(0.376 to 1.460) | 0.789(0.395 to 1.578) | Fixed | 0.0000 | 0.364 | 0.386 | 0.503 | 0.959 to 5.418 | 0.0 | 0.379 | NA | No | NA | V | Low |
| IC (>2w)^12^ | esophageal cancer | 99/566 | 4 | 0 | 4 | ***0.334(0.166 to 0.670)*** | 0.294(0.115 to 0.751) | 0.175(0.044 to 0.701) | Fixed | 0.2797 | 0.002 | 0.011 | 0.014 | 0.263 to 19.802 | 30.0 | 0.232 | 0.143 | No | No | IV | Low |
| nCRT^83^ | esophageal cancer | 142/1515 | 11 | 0 | 11 | *1.037(0.724 to 1.486)* | 1.020(0.707 to 1.472) | 0.784(0.479 to 1.285) | Fixed | 0.0000 | 0.844 | 0.917 | 0.334 | 1.024 to 5.077 | 0.0 | 0.902 | 0.016 | Yes | Yes | V | Low |
| nCRT>7-8w^84^ | esophageal cancer | 467/4198 | 6 | 0 | 6 | *1.120(0.912 to 1.376)* | 1.136(0.730 to 1.769) | 1.090(0.857 to 1.388) | Fixed | 0.1290 | 0.278 | 0.571 | 0.482 | 0.899 to 5.779 | 49.7 | 0.077 | 0.656 | Yes | Yes | V | Low |
| nCRT≤7-8w^85^ | esophageal cancer | 39/928 | 4 | 0 | 4 | *0.754(0.395 to 1.439)* | 0.779(0.403 to 1.505) | 0.227(0.025 to 2.088) | Fixed | 0.0000 | 0.392 | 0.457 | 0.190 | 0.143 to 36.229 | 0.0 | 0.637 | 0.015 | No | Yes | V | Low |
| nCT^83^ | esophageal cancer | 93/1526 | 8 | 0 | 8 | *0.994(0.651 to 1.515)* | 0.968(0.628 to 1.493) | 0.942(0.527 to 1.683) | Fixed | 0.0000 | 0.976 | 0.884 | 0.841 | 1.103 to 4.711 | 0.0 | 0.784 | 0.667 | Yes | Yes | V | Very Low |
| SDD^13^ | esophageal and gastric cancer | 40/415 | 4 | 4 | 0 | ***0.388(0.188 to 0.801)*** | 0.397(0.191 to 0.825) | 0.253(0.069 to 0.937) | Fixed | 0.0000 | 0.010 | 0.013 | 0.040 | 0.447 to 11.641 | 0.0 | 0.866 | 0.804 | No | No | IV | Low |
| **Intraoperative operations** | |  |  |  |  |  |  |  |  |  |  |  |  |  |  |  |  |  |  |  |  |
| TTE vs THE^5^ | esophageal cancer | 1782/17670 | 17 | 0 | 17 | 0.864(0.778 to 0.959) | ***0.719(0.561 to 0.920)*** | 0.187(0.082 to 0.425) | Random | 0.1179 | 0.006 | 0.009 | 0.000 | 0.604 to 8.609 | 68.0 | 0.000 | 0.061 | Yes | Yes | IV | Low |
| Stapled (vs hand-sewn)^14^ | esophageal cancer | NR/611 | 6 | 6 | 0 | ***0.546(0.209 to 0.884)*** | 0.546(0.209 to 0.884) | 0.400(0.170 to 0.950) | Fixed | 0.0000 | 0.002 | 0.002 | 0.044 | 0.778 to 6.683 | 0.0 | 0.696 | 0.625 | Yes | No | IV | Low |
| Omentoplasty^5^ | esophageal cancer | 119/1487 | 7 | 0 | 7 | 0.344(0.221 to 0.536) | ***0.281(0.131 to 0.602)*** | 0.017(0.001 to 0.356) | Random | 0.4684 | 0.000 | 0.001 | 0.009 | 0.040 to 129.121 | 51.1 | 0.056 | 0.026 | Yes | Yes | IV | Low |
| Thoracic (vs cervical)^5^ | esophageal cancer | 1277/10093 | 14 | 0 | 14 | 1.550(1.366 to 1.758) | ***2.084(1.561 to 2.781)*** | 1.143(0.919 to 1.422) | Random | 0.1313 | 0.000 | 0.000 | 0.230 | 0.901 to 5.771 | 67.2 | 0.000 | 0.014 | No | Yes | III | Low |
| TAFT^89^ | Esophageal atresia | 75/455 | 4 | 0 | 4 | *1.801(0.933 to 3.473)* | 1.791(0.928 to 3.458) | 1.647(0.732 to 3.705) | Fixed | 0.0000 | 0.079 | 0.083 | 0.227 | 0.827 to 6.282 | 0.0 | 0.954 | 0.455 | No | No | V | Low |
| ICG^15^ | esophageal cancer | 150/1162 | 9 | 0 | 9 | ***0.252(0.152 to 0.418)*** | 0.276(0.161 to 0.472) | 0.274(0.112 to 0.667) | Fixed | 0.0000 | 0.000 | 0.000 | 0.004 | 0.748 to 6.954 | 0.0 | 0.452 | 0.888 | Yes | No | II | Very Low |
| Injection botox^92^ | esophageal cancer | 85/442 | 4 | 0 | 4 | 1.123(0.641 to 1.970) | *1.199(0.432 to 3.331)* | 0.620(0.267 to 1.436) | Random | 0.5106 | 0.685 | 0.728 | 0.489 | 0.296 to 17.573 | 52.0 | 0.100 | 0.952 | No | Yes | V | Low |
| FJT^90^ | locally advanced esophageal cancer | 382/4101 | 16 | 0 | 16 | ***1.274(1.022 to 1.590)*** | 1.233(0.984 to 1.546) | 1.063(0.803 to 1.407) | Fixed | 0.0000 | 0.032 | 0.069 | 0.668 | 1.606 to 3.237 | 0.0 | 0.603 | 0.382 | No | No | V | Low |
| Hypotension^5^ | esophageal cancer | 236/2637 | 4 | 0 | 4 | 1.975(1.467 to 2.659) | ***4.344(1.189 to 15.864)*** | 1.398(0.986 to 1.982) | Random | 1.3855 | 0.000 | 0.026 | 0.060 | 0.123 to 42.133 | 84.9 | 0.000 | 0.181 | Yes | No | IV | Very Low |
| Blood transfusion^5^ | esophageal cancer | 1124/10917 | 6 | 0 | 6 | ***1.211(1.013 to 1.448)*** | 1.210(1.011 to 1.449) | 1.196(0.947 to 1.511) | Fixed | 0.0000 | 0.036 | 0.038 | 0.133 | 1.703 to 3.053 | 0.0 | 0.680 | 0.661 | No | No | IV | Very Low |
| Mechanical stretching^8^ | esophageal cancer | 19/267 | 4 | 0 | 4 | *0.577(0.201 to 1.652)* | 0.619(0.215 to 1.782) | 0.549(0.153 to 1.964) | Fixed | 0.0000 | 0.305 | 0.374 | 0.356 | 0.463 to 11.239 | 0.0 | 0.474 | 0.998 | Yes | Yes | V | Very Low |
| Steroids^5^ | esophageal cancer | 791/7307 | 3 | 0 | 3 | ***1.886(1.294 to 2.748)*** | 1.857(1.269 to 2.717) | 1.744(1.094 to 2.781) | Fixed | 0.0000 | 0.001 | 0.001 | 0.019 | 1.273 to 4.085 | 0.0 | 0.586 | 0.921 | No | No | IV | Very Low |
| **Postoperative management** |  |  |  |  |  |  |  |  |  |  |  |  |  |  |  |  |  |  |  |  |  |
| Early removal of nasogastric tubes^95^ | esophageal cancer | 48/608 | 7 | 4 | 3 | *0.721(0.389 to 1.337)* | 0.820(0.427 to 1.575) | 0.069(0.004 to 1.313) | Fixed | 0.0000 | 0.299 | 0.551 | 0.075 | 0.061 to 85.224 | 0.0 | 0.527 | 0.043 | Yes | Yes | V | Low |
| Pyloric balloon dilatation^96^ | esophageal cancer | 17/191 | 2 | 0 | 2 | *0.950(0.317 to 2.846)* | 0.948(0.316 to 2.846) | 0.905(0.261 to 3.145) | Fixed | 0.0000 | 0.927 | 0.925 | 0.876 | 0.481 to 10.803 | 0.0 | 0.877 | NA | No | NA | V | Very Low |

**Abbreviations:** ASA, American Society of Anaesthesiologist; BMI, body mass index; COPD, chronic obstructive pulmonary disease; FEV1, forced expiratory volume in 1 second; ThAO, thoracic aorta; CA, celiac axis; RPCA, right post-celiac axis; LPCA, left post-celiac axis; LGA, left gastric artery; SGA, short gastric arteries; SA, splenic artery; IC, ischemic conditioning; nCRT, neoadjuvant chemoradiotherapy; nCT, neoadjuvant chemotherapy; SDD, selective decontam ination of the digestive tract; TTE, transthoracic esophagectomy; THE, transhiatal esophagectomy; TAFT, transanastomotic feeding tube; ICG, Indocyanine green; FJT, feeding jejunostomy tube; OR, odds ratio; NA, not available; NR, not reported; RCT, randomized controlled study; OE, observational study; AMSTAR 2, a measurement tool to assess systematic reviews; CI, confidence interval.
