# Supplementary material for: Risk and protective factors for postoperative anastomotic leakage in esophageal and gastrointestinal surgery: an umbrella review of meta-analyses and systematic reviews
Source: Int J Surg. 2025 Sep 19;112(1):1722–36. doi: 10.1097/JS9.0000000000003308 (PMC12825836; doi:10.1097/JS9.0000000000003308)
Supplement: Supplementary file 4 [file js9-112-1722-005.docx]

| **1Factors** | **Patients** | E**vents or cases/total(n.)** | **Studies(n.)** | | | **Summary relative risk estimate(OR,95% CI)** | | | **Selected effect model** | **Tau^2^** | **P value** | | | **95% PI** | **Heterogeneity** | | **Egger's**  **P value** | **Excess**  **significance** | **Small-study effect** | **Evidence class** | **AMSTAR 2** |
| --- | --- | --- | --- | --- | --- | --- | --- | --- | --- | --- | --- | --- | --- | --- | --- | --- | --- | --- | --- | --- | --- |
|  |  |  | **Total** | **RCT** | **OE** | **Fixed effects** | **Random effects** | **Largest study** |  |  | **Fixed** | **Random** | **Largest** |  | **I^2^(%)** | **I^2^ -P value** |  |  |  |  |  |
| **Various factors vs. placebo or conventional treatment** | | |  |  |  |  |  |  |  |  |  |  |  |  |  |  |  |  |  |  |  |
| **Patient characteristics** | |  |  |  |  |  |  |  |  |  |  |  |  |  |  |  |  |  |  |  |  |
| BMI ≥25^80^ | GC | 322/12367 | 19 | 0 | 19 | ***0.565(0.443 to 0.722)*** | 0.577(0.404 to 0.825) | 0.153(0.060 to 0.391) | Fixed | 0.1866 | 0.000 | 0.003 | 0.000 | 0.470 to 11.055 | 34.7 | 0.069 | 0.333 | Yes | Yes | IV | Low |
| **Intraoperative operations** |  |  |  |  |  |  |  |  |  |  |  |  |  |  |  |  |  |  |  |  |  |
| PPG^86^ | Esophageal neoplasms | NR | 10 | 0 | 10 | ***0.295(-0.147 to 0.737)*** | 0.295(-0.147 to 0.737) | 0.200(0.040 to 1.120) | Fixed | 0.0000 | 0.191 | 0.191 | 0.468 | 0.284 to 18.298 | 0.0 | 1.000 | 0.994 | No | No | V | Low |
| Pouch Roux-en-Y^87^ | GC | 30/644 | 8 | 8 | 0 | *1.731(0.778 to 3.853)* | 1.567(0.665 to 3.692) | 1.291(0.423 to 3.939) | Fixed | 0.0000 | 0.179 | 0.305 | 0.654 | 0.565 to 9.196 | 0.0 | 0.611 | 0.977 | Yes | No | V | Low |
| Aboral pouch^88^ | gastric carcinomas | 21/363 | 2 | 2 | 0 | 0.245(0.026 to 2.272) | 0.245(0.026 to 2.272) | 0.245(0.026 to 2.272) | NA | NA | 0.216 | 0.216 | 0.216 | NA | NA | NA | NA | Yes | NA | V | Low |
| Jejunal pouch^88^ | gastric carcinomas | 40/1235 | 11 | 8 | 3 | *1.841(0.950 to 3.565)* | 1.744(0.754 to 4.036) | 0.263(0.025 to 2.793) | Fixed | 0.1922 | 0.070 | 0.194 | 0.268 | 0.099 to 52.524 | 14.6 | 0.318 | 0.880 | Yes | No | V | Low |
| Splenectomy^91^ | GC | 47/1009 | 7 | 1 | 6 | ***1.822(1.010 to 3.290)*** | 1.813(0.981 to 3.350) | 0.705(0.167 to 2.970) | Fixed | 0.0000 | 0.046 | 0.058 | 0.633 | 0.377 to 13.779 | 0.0 | 0.609 | 0.862 | Yes | No | V | Low |
| Preduodenal pouch^88^ | GC | 2/60 | 1 | 1 | 0 | 1.000(0.060 to 16.763) | 1.000(0.060 to 16.763) | 1.000(0.060 to 16.763) | NA | NA | 1.000 | 1.000 | 1.000 | NA | NA | NA | NA | Yes | NA | V | Low |
| Seprafilm^56^ | gastrointestinal neoplasms | 40/1731 | 5 | 2 | 3 | *1.256(0.669 to 2.358)* | 1.238(0.645 to 2.374) | 0.694(0.170 to 2.833) | Fixed | 0.0000 | 0.478 | 0.521 | 0.610 | 0.393 to 13.230 | 0.0 | 0.655 | 0.265 | Yes | No | V | Low |
| Sealant^16^ | gastrointestinal diseases | 230/3024 | 14 | 5 | 9 | ***0.374(0.271 to 0.517)*** | 0.394(0.283 to 0.547) | 0.197(0.066 to 0.583) | Fixed | 0.0000 | 0.000 | 0.000 | 0.003 | 0.584 to 8.897 | 0.0 | 0.835 | 0.038 | No | Yes | II | Low |
| Without drian^93^ | GC | 78/4375 | 15 | 4 | 11 | *0.867(0.535 to 1.405)* | 0.902(0.550 to 1.479) | 0.194(0.022 to 1.685) | Fixed | 0.0000 | 0.562 | 0.683 | 0.137 | 0.151 to 34.320 | 0.0 | 0.890 | 0.207 | Yes | Yes | V | Low |
| **Postoperative management** | |  |  |  |  |  |  |  |  |  |  |  |  |  |  |  |  |  |  |  |  |
| Rapid rehabilitation^73^ | gastrointestinal diseases | 180/3378 | 19 | NR | NR | *0.896(0.658 to 1.221)* | 0.889(0.648 to 1.221) | 0.728(0.392 to 1.352) | Fiexd | 0.0000 | 0.487 | 0.469 | 0.315 | 1.052 to 4.943 | 0.0 | 0.990 | 0.789 | Yes | Yes | V | Very Low |
| NSAIDs^21^ | gastrointestinal diseases | 1922/31876 | 24 | 6 | 18 | 1.262(1.142 to 1.396) | ***1.688(1.278 to 2.230)*** | 1.145(0.946 to 1.385) | Random | 0.2635 | 0.000 | 0.000 | 0.164 | 0.634 to 8.200 | 79.1 | 0.000 | 0.027 | No | Yes | III | Very Low |
| Non-selective NSAIDs^21^ | gastrointestinal diseases | 864/10424 | 15 | 5 | 10 | 1.222(1.054 to 1.416) | ***1.805(1.119 to 2.910)*** | 0.529(0.388 to 0.722) | Random | 0.5429 | 0.008 | 0.015 | 0.000 | 0.360 to 14.449 | 85.1 | 0.000 | 0.132 | No | Yes | IV | Very Low |
| Non-systematic NSAIDs^21^ | gastrointestinal diseases | 1831/29958 | 13 | 0 | 13 | 1.174(1.058 to 1.302) | ***1.344(1.033 to 1.749)*** | 0.621(0.472 to 0.816) | Random | 0.1738 | 0.003 | 0.028 | 0.001 | 0.776 to 6.695 | 81.1 | 0.000 | 0.173 | Yes | Yes | IV | Very Low |
| Elective COX-2 NSAIDs^21^ | gastrointestinal diseases | 416/4404 | 8 | 2 | 6 | 1.321(1.010 to 1.727) | *1.675(0.896 to 3.130)* | 0.908(0.614 to 1.343) | Random | 0.4219 | 0.042 | 0.106 | 0.630 | 0.431 to 12.050 | 66.5 | 0.004 | 0.394 | Yes | No | V | Very Low |
| Protocol-based NSAIDs^21^ | gastrointestinal diseases | 89/1918 | 11 | 6 | 5 | ***4.507(2.798 to 7.260)*** | 4.254(2.437 to 7.425) | 0.872(0.156 to 4.883) | Fiexd | 0.1159 | 0.000 | 0.000 | 0.877 | 0.227 to 22.930 | 14.7 | 0.307 | 0.052 | Yes | Yes | III | Very Low |
| Decompression^94^ | GC | 40/1141 | 8 | 8 | 0 | *1.261(0.671 to 2.370)* | 1.250(0.662 to 2.360) | 1.170(0.412 to 3.323) | Fiexd | 0.0000 | 0.472 | 0.492 | 0.768 | 0.618 to 8.406 | 0.0 | 0.997 | 0.450 | Yes | No | V | Very Low |
| Early Oral Feeding^20^ | GC | 1/454 | 7 | 7 | 0 | 0.298(0.012 to 7.653) | 0.298(0.012 to 7.653) | NA | NA | NA | 0.465 | 0.465 | NA | NA | NA | NA | NA | Yes | NA | V | Low |
| Early feeding^79^ | gastrointestinal diseases | 29/1075 | 13 | 13 | 0 | *0.709(0.350 to 1.438)* | 0.694(0.328 to 1.467) | 0.475(0.086 to 2.630) | Fiexd | 0.0000 | 0.340 | 0.339 | 0.394 | 0.269 to 19.335 | 0.0 | 0.903 | 0.211 | Yes | No | V | Low |
| NACS^22^ | AGC | 98/2567 | 16 | 5 | 11 | ***0.513(0.319 to 0.826)*** | 0.545(0.333 to 0.890) | 0.341(0.098 to 1.185) | Fiexd | 0.0000 | 0.006 | 0.015 | 0.091 | 0.480 to 10.827 | 0.0 | 0.977 | 0.881 | No | No | IV | Low |
| **Perioperative operation** |  |  |  |  |  |  |  |  |  |  |  |  |  |  |  |  |  |  |  |  |  |
| NSAIDs^75^ | Gastrointestinal diseases | 157/2236 | 10 | 6 | 4 | ***3.018(2.155 to 4.227)*** | 3.672(1.935 to 6.969) | 1.849(1.159 to 2.951) | Fiexd | 0.3719 | 0.000 | 0.000 | 0.010 | 0.458 to 11.341 | 49.7 | 0.044 | 0.502 | Yes | No | II | Low |
| Non-selective NSAIDs^75^ | Gastrointestinal diseases | 112/1466 | 8 | 5 | 3 | ***2.964(1.990 to 4.415)*** | 3.385(1.688 to 6.789) | 2.037(1.211 to 3.427) | Fiexd | 0.2870 | 0.000 | 0.001 | 0.007 | 0.527 to 9.864 | 37.6 | 0.142 | 0.570 | Yes | No | IV | Low |
| Selective NSAIDs^75^ | Gastrointestinal diseases | 76/1264 | 4 | 2 | 2 | 2.574(1.547 to 4.282) | *2.269(0.682 to 7.556)* | 1.175(0.504 to 2.741) | Random | 0.8978 | 0.000 | 0.182 | 0.709 | 0.178 to 29.239 | 69.1 | 0.021 | 0.757 | No | No | V | Low |

**Table S3. Characteristics and quality assessment of the meta-analyses investigating protective and dangerous factors concerning gastric cancer. Associations reported in italic are those retained in the main analysis. Furthermore, significant associations(P<0.05) are presented in bold.**

**Abbreviations:** BMI, body mass index; PPG, pylorus-preserving gastrectomy; NSAIDs, non-steroidal anti-inflammatory drugs; NACS, neoadjuvant chemotherapy followed by surgery; AGC, advanced gastric cancer; GC, gastric cancer; GD, Gastrointestinal diseases; NR, not reported; NA, not available; RCT, randomized controlled study; OE, observational study; AMSTAR 2, a measurement tool to assess systematic reviews; CI, confidence interval.
