# Supplementary material for: Risk and protective factors for postoperative anastomotic leakage in esophageal and gastrointestinal surgery: an umbrella review of meta-analyses and systematic reviews
Source: Int J Surg. 2025 Sep 19;112(1):1722–36. doi: 10.1097/JS9.0000000000003308 (PMC12825836; doi:10.1097/JS9.0000000000003308)
Supplement: Supplementary file 5 [file js9-112-1722-006.docx]

| **0Factors**  **Table S4. Characteristics and quality assessment of the meta-analyses investigating protective and dangerous factors concerning colorectal cancer (CRC). Associations reported in italic are those retained in the main analysis. Furthermore, significant associations(P<0.05) are presented in bold.** | **Patients** | E**vents or cases/total(n.)** | **Studies(n.)** | | | **Summary odds ratio estimate(OR,95% CI)** | | | **Selected effect model** | **Tau^2^** | **P value** | | | **95% PI** | **Heterogeneity** | | **Egger's**  **P value** | **Excess**  **significance** | **Small-study effect** | **Evidence class** | **AMSTAR 2** |
| --- | --- | --- | --- | --- | --- | --- | --- | --- | --- | --- | --- | --- | --- | --- | --- | --- | --- | --- | --- | --- | --- |
|  |  |  | **Total** | **RCT** | **OE** | **Fixed effects** | **Random effects** | **Largest study** |  |  | **Fixed** | **Random** | **Largest** |  | **I^2^(%)** | **I^2^ -P value** |  |  |  |  |  |
| **Various factors vs. placebo or conventional treatment** | | |  |  |  |  |  |  |  |  |  |  |  |  |  |  |  |  |  |  |  |
| **Patient characteristics** | |  |  |  |  |  |  |  |  |  |  |  |  |  |  |  |  |  |  |  |  |
| ASA (3-4)^4^ | CRC | 2444/34932 | 16 | 0 | 16 | 1.386(1.261 to 1.523) | ***1.762 (1.377 to 2.254)*** | 1.326 (1.162 to 1.514) | Random | 0.1008 | 0.000 | 0.000 | 0.000 | 1.029 to 5.050 | 64.0 | 0.000 | 0.100 | No | Yes | II | Low |
| BMI>30^45^ | CRC (Western) colonic and rectal anastomoses | 1640/21453 | 13 | 0 | 13 | ***1.215 (1.062 to 1.390)*** | 1.402 (1.065 to 1.844) | 1.073 (0.901 to 1.276) | Fixed | 0.0941 | 0.005 | 0.016 | 0.429 | 1.043 to 4.986 | 49.6 | 0.022 | 0.393 | Yes | No | IV | Low |
| BMI>25^45^ | CRC (Asian) colonic and rectal anastomoses | 580/11500 | 18 | 0 | 18 | ***1.286 (1.070 to 1.546)*** | 1.426 (1.122 to 1.813) | 0.995 (0.662 to 1.494) | Fixed | 0.0606 | 0.007 | 0.004 | 0.979 | 1.036 to 5.019 | 25.6 | 0.154 | 0.027 | Yes | Yes | IV | Low |
| Visceral obesity^47^ | CRC | 29/526 | 3 | 0 | 3 | ***2.404(1.063 to 5.438)*** | 2.247(0.555 to 9.096) | 0.255(0.013 to 4.954) | Fixed | 0.7095 | 0.035 | 0.256 | 0.367 | 0.033 to 159.706 | 47.1 | 0.151 | 0.546 | Yes | No | V | Low |
| Diabetes mellitus^4^ | CRC | 861/11871 | 16 | 0 | 16 | 1.729(1.455 to 2.054) | ***2.138(1.490 to 3.067)*** | 1.175(0.886 to 1.558) | Random | 0.3370 | 0.000 | 0.000 | 0.262 | 0.527 to 9.870 | 68.0 | 0.000 | 0.253 | No | No | III | Low |
| Sarcopenia^47^ | CRC | 185/3189 | 12 | 0 | 12 | *0.991(0.723 to 1.358)* | 1.358(0.721 to 1.374) | 0.589(0.332 to 1.045) | Fixed | 0.0000 | 0.955 | 0.975 | 0.070 | 1.114 to 4.668 | 0.0 | 0.475 | 0.406 | Yes | Yes | V | Low |
| Old age^4^ | CRC | NA/17493 | 7 | 0 | 7 | ***0.990(0.985 to 0.995)*** | 0.941(0.848 to 1.044) | 0.990(0.985 to 0.995) | Fixed | 0.0056 | 0.000 | 0.254 | 0.000 | 1.898 to 2.739 | 36.3 | 0.152 | 0.255 | No | Yes | V | Low |
| Male subjects^4^ | CRC | NA/93016 | 11 | 0 | 11 | ***1.478(1.365 to 1.601)*** | 1.478(1.365 to 1.601) | 1.490(1.352 to 1.642) | Fixed | 0.0000 | 0.000 | 0.000 | 0.000 | 2.019 to2.574 | 0.0 | 0.883 | 0.742 | No | Yes | II | Low |
| **Intraoperative operations** | |  |  |  |  |  |  |  |  |  |  |  |  |  |  |  |  |  |  |  |  |
| Air leak test (+)^48^ | CRC | 155/2887 | 9 | 1 | 8 | 0.797(0.564 to 1.127) | *0.613(0.317 to 1.185)* | 0.488(0.256 to 0.931) | Random | 0.4494 | 0.199 | 0.146 | 0.029 | 0.366 to 14.212 | 55.3 | 0.022 | 0.160 | Yes | Yes | V | Low |
| LCA preservation^49^ | RC | 106/1587 | 10 | 7 | 3 | ***0.430(0.289 to 0.640)*** | 0.456(0.304 to 0.685) | 0.525(0.333 to 0.827) | Fixed | 0.0000 | 0.000 | 0.000 | 0.005 | 1.291 to 4.026 | 0.0 | 0.937 | 0.006 | No | Yes | III | Low |
| IMA preserving^50^ | diverticular disease | 39/400 | 4 | 1 | 3 | 0.487(0.248 to 0.959) | *0.720(0.109 to 4.763)* | 0.222(0.088 to 0.554) | Random | 2.4633 | 0.037 | 0.733 | 0.001 | 0.041 to 126.176 | 72.7 | 0.012 | 0.597 | Yes | Yes | V | Low |
| IMA high ligation^51^ | sigmoid colon and rectal cancer | 474/5917 | 18 | 4 | 14 | ***1.325(1.090 to 1.610)*** | 1.317(1.081 to 1.603) | 1.011(0.746 to 1.369) | Fixed | 0.0000 | 0.005 | 0.006 | 0.946 | 1.560 to 3.332 | 0.0 | 0.751 | 0.021 | Yes | Yes | IV | Low |
| Low anastomosis^52^ | CRC | NA/7175 | 6 | 0 | 6 | ***3.265(2.314 to 4.608)*** | 3.265(2.305 to 2.305) | 2.490(1.371 to 4.523) | Fixed | 0.0028 | 0.000 | 0.000 | 0.003 | 1.069 to 4.862 | 1.4 | 0.407 | 0.417 | No | No | I | Low |
| End-to-side//J-pouch^7^ | RC | 777/7419 | 10 | 0 | 10 | 0.888(0.742 to 1.063) | *0.871(0.651 to 1.165)* | 0.619(0.379 to 1.014) | Random | 0.0946 | 0.196 | 0.353 | 0.057 | 0.862 to 6.031 | 50.6 | 0.033 | 0.214 | No | Yes | V | Low |
| Ileostomy^53^ | RC | 92/764 | 5 | 5 | 0 | ***0.286(0.174 to 0.470)*** | 0.292(0.177 to 0.481) | 0.309(0.150 to 0.636) | Fixed | 0.0000 | 0.000 | 0.000 | 0.001 | 0.924 to 5.624 | 0.0 | 0.797 | 0.467 | No | No | IV | Low |
| LI (loop ileostomy)^58^ | RC | 14/994 | 5 | 2 | 3 | *1.420(0.517 to 3.897)* | 0.929(0.162 to 5.337) | 0.101(0.005 to 2.135) | Fixed | 1.4426 | 0.496 | 0.934 | 0.141 | 0.519 to 275.579 | 36.3 | 0.179 | 0.382 | Yes | Yes | V | Low |
| Emergency surgery^4^ | CRC | 1960/29546 | 5 | 0 | 5 | 1.509(1.316 to 1.729) | ***1.675(1.285 to 2.184)*** | 1.432(1.200 to 1.709) | Random | 0.0441 | 0.000 | 0.000 | 0.000 | 1.302 to 3.991 | 56.1 | 0.058 | 0.073 | No | Yes | II | Low |
| TDT^9^ | RC | 113/1385 | 5 | 5 | 0 | 0.892(0.607 to 1.312) | *0.906(0.491 to 1.674)* | 0.393(0.168 to 0.919) | Random | 0.2451 | 0.562 | 0.753 | 0.031 | 0.455 to 11.432 | 52.4 | 0.078 | 0.856 | Yes | Yes | V | Low |
| TDT (Grade B)^9^ | RC | 46/1115 | 3 | 3 | 0 | *1.194(0.659 to 2.164)* | 1.190(0.651 to 2.176) | 0.685(0.208 to 2.259) | Fixed | 0.0000 | 0.559 | 0.572 | 0.535 | 0.513 to 10.124 | 0.0 | 0.497 | 0.976 | No | Yes | V | Low |
| TDT (Grade C)^9^ | RC | 32/1115 | 3 | 3 | 0 | ***0.270(0.116 to 0.628)*** | 0.315(0.097 to 1.022) | 0.115(0.026 to 0.507) | Fixed | 0.3794 | 0.002 | 0.054 | 0.252 | 0.208 to 24.945 | 34.4 | 0.218 | 0.846 | Yes | No | V | Low |
| TDT (with a stoma)^9^ | RC | 22/250 | 2 | 2 | 0 | ***2.630(1.028 to 6.726)*** | 2.591(1.003 to 6.692) | 1.932(0.524 to 7.127) | Fixed | 0.0000 | 0.044 | 0.049 | 0.323 | 0.446 to 11.653 | 0.0 | 0.520 | NA | No | Yes | IV | Low |
| Drain (intra)^54^ | CRD | 32/951 | 5 | 5 | 0 | *1.117(0.548 to 2.277)* | 1.103(0.523 to 2.329) | 1.242(0.452 to 3.410) | Fixed | 0.0000 | 0.762 | 0.797 | 0.674 | 0.645 to 8.062 | 0.0 | 0.660 | 0.765 | Yes | Yes | V | Low |
| Drain (extra)^54^ | CRD | 36/281 | 3 | 3 | 0 | *1.094(0.541 to 2.210)* | 1.095(0.539 to 2.221) | 1.109(0.390 to 3.155) | Fixed | 0.0000 | 0.803 | 0.802 | 0.846 | 0.617 to 8.422 | 0.0 | 0.859 | 0.534 | No | No | V | Low |
| Drain (intra and extra)^54^ | CRD | 49/561 | 4 | 4 | 0 | *1.395(0.731 to 2.665)* | 1.392(0.727 to 2.664) | 1.426(0.601 to 3.383) | Fixed | 0.0000 | 0.313 | 0.318 | 0.421 | 0.774 to 6.714 | 0.0 | 0.982 | 0.501 | No | No | V | Low |
| Drain (Active)^54^ | CRD | 83/798 | 4 | 4 | 0 | *1.149(0.716 to 1.844)* | 1.150(0.713 to 1.855) | 1.133(0.553 to 2.323) | Fixed | 0.0000 | 0.566 | 0.566 | 0.733 | 0.930 to 5.591 | 0.0 | 0.689 | 0.411 | No | No | V | Low |
| Drain (Passive)^54^ | CRD | 30/688 | 6 | 6 | 0 | *1.384(0.650 to 2.947)* | 1.339(0.615 to 2.917) | 1.186(0.356 to 3.946) | Fixed | 0.0000 | 0.399 | 0.462 | 0.781 | 0.507 to 10.254 | 0.0 | 0.922 | 0.549 | No | No | V | Low |
| Drain (Active and Passive)^54^ | CRD | 4/317 | 1 | 1 | 0 | 0.340(0.035 to 3.302) | 0.340(0.035 to 3.302) | NA | NA | NA | 0.352 | 0.352 | NA | NA | NA | NA | NA | No | NA | V | Low |
| Drain (clinnical ASL)^54^ | CRD | 51/1493 | 9 | 9 | 0 | *1.414(0.792 to 2.526)* | 1.402(0.775 to 2.536) | 1.186(0.356 to 3.946) | Fixed | 0.0000 | 0.241 | 0.263 | 0.781 | 0.507 to 10.254 | 0.0 | 0.985 | 0.542 | Yes | No | V | Low |
| Drain (radiologic ASL)^54^ | CRD | 60/1288 | 7 | 7 | 0 | *0.914(0.538 to 1.554)* | 0.918(0.530 to 1.591) | 0.905(0.392 to 2.092) | Fixed | 0.0000 | 0.740 | 0.761 | 0.815 | 0.801 to 6.494 | 0.0 | 0.798 | 0.344 | No | No | V | Low |
| SFM (HRR)^8^ | CRC | 17/979 | 4 | 0 | 4 | ***2.741(1.041 to 7.218)*** | 2.933(1.084 to 7.931) | 0.471(0.018 to 12.406) | Fixed | 0.0000 | 0.041 | 0.034 | 0.652 | 0.038 to 135.491 | 0.0 | 0.687 | 0.141 | No | No | IV | Very Low |
| SFM (LRR)^8^ | CRC | 83/754 | 7 | 0 | 7 | *1.551(0.913 to 2.635)* | 1.470(0.855 to 2.527) | 1.146(0.344 to 3.814) | Fixed | 0.0000 | 0.105 | 0.164 | 0.824 | 0.507 to 10.255 | 0.0 | 0.884 | 0.500 | No | No | V | Very Low |
| SFM (HRR+LRR)^8^ | CRC | 1266/40488 | 6 | 0 | 6 | *0.997(0.890 to 1.117)* | 0.998(0.891 to 1.119) | 0.990(0.874 to 1.122) | Fixed | 0.0000 | 0.963 | 0.975 | 0.879 | 1.950 to 2.665 | 0.0 | 0.572 | 0.756 | No | Yes | V | Very Low |
| SFM (Laparotomic, LT)^8^ | CRC | 109/1705 | 5 | 0 | 5 | *1.413(0.945 to 2.114)* | 1.417(0.946 to 2.124) | 1.438(0.904 to 2.288) | Fixed | 0.0000 | 0.092 | 0.091 | 0.125 | 1.276 to 4.074 | 0.0 | 0.652 | 0.933 | No | Yes | V | Very Low |
| SFM (Laparoscopic, LS)^8^ | CRC | 58/676 | 5 | 0 | 5 | ***1.910(1.003 to 3.638)*** | 1.794(0.764 to 4.212) | 0.333(0.033 to 3.346) | Fixed | 0.2316 | 0.049 | 0.179 | 0.350 | 0.101 to 51.550 | 24.4 | 0.259 | 0.598 | Yes | No | V | Very Low |
| SFM (LT+LS)^8^ | CRC | 1198/39810 | 5 | 0 | 5 | *0.985(0.877 to 1.107)* | 0.986(0.877 to 1.108) | 0.990(0.874 to 1.122) | Fixed | 0.0000 | 0.806 | 0.813 | 0.879 | 1.950 to 2.665 | 0.0 | 0.921 | 0.363 | No | No | V | Very Low |
| SFM (HRR in LT)^8^ | CRC | 9/751 | 2 | 0 | 2 | *2.255(0.594 to 8.555)* | 2.331(0.527 to 10.308) | 1.000(0.128 to 7.812) | Fixed | 0.1946 | 0.232 | 0.265 | 1.000 | 0.140 to 37.034 | 16.7 | 0.273 | NA | No | NA | V | Very Low |
| SFM (LRR in LT)^8^ | CRC | 14/132 | 2 | 0 | 2 | *1.028(0.326 to 3.238)* | 1.001(0.322 to 3.110) | 1.1469(0.344 to 3.814) | Fixed | 0.0000 | 0.963 | 0.998 | 0.824 | 0.507 to 10.255 | 0.0 | 0.511 | NA | No | NA | V | Very Low |
| SFM (LRR+HRR in LT)^8^ | CRC | 86/882 | 2 | 0 | 2 | *1.415(0.899 to 2.228)* | 1.415(0.898 to 2.231) | 1.438(0.904 to 2.288) | Fixed | 0.0000 | 0.134 | 0.135 | 0.125 | 1.276 to 4.074 | 0.0 | 0.728 | NA | No | NA | V | Very Low |
| SFM (HRR in LS)^8^ | CRC | 7/193 | 2 | 0 | 2 | *4.315(0.770 to 24.161)* | 4.699(0.876 to 25.208) | 2.727(0.275 to 27.076) | Fixed | 0.0000 | 0.096 | 0.071 | 0.392 | 0.129 to 40.153 | 0.0 | 0.491 | NA | No | NA | V | Very Low |
| SFM (LRR in LS)^8^ | CRC | 37/275 | 3 | 0 | 3 | *2.239(0.832 to 6.027)* | 2.085(0.574 to 7.574) | 1.136(0.292 to 4.430) | Fixed | 0.3283 | 0.111 | 0.264 | 0.854 | 0.252 to 20.668 | 37.7 | 0.205 | NA | Yes | NA | V | Very Low |
| SFM (LRR + HRR in LS)^8^ | CRC | 14/208 | 2 | 0 | 2 | *0.552(0.148 to 2.065)* | 0.558(0.150 to 2.084) | 0.333(0.033 to 3.346) | Fixed | 0.0000 | 0.378 | 0.386 | 0.350 | 0.127 to 40.897 | 0.0 | 0.593 | NA | Yes | NA | V | Very Low |
| Hand sewn^7^ | RC | 2417/20395 | 10 | 0 | 10 | 0.876(0.739 to 1.039) | *0.739(0.456 to 1.198)* | 0.119(0.051 to 0.278) | Random | 0.3069 | 0.128 | 0.220 | 0.000 | 0.407 to 12.759 | 64.6 | 0.003 | 0.484 | Yes | Yes | V | Low |
| Long Operation time^7^ | RC | 90/1594 | 3 | 0 | 3 | 1.230(0.796 to 1.900) | *0.871(0.256 to 2.965)* | 0.229(0.063 to 0.826) | Random | 0.9287 | 0.351 | 0.826 | 0.024 | 0.131 to 39.687 | 80.8 | 0.005 | 0.186 | Yes | Yes | V | Low |
| IORT (vs surgery/EBRT)^97^ | RC | 36/591 | 5 | NR | NR | *1.032(0.519 to 2.050)* | 1.057(0.512 to 2.183) | 0.390(0.100 to 1.523) | Fixed | 0.0000 | 0.929 | 0.880 | 0.175 | 0.416 to 12.506 | 0.0 | 0.510 | 0.869 | No | Yes | V | Very Low |
| IORT^55^ | RC | NR | 7 | 3 | 4 | ***0.462(-0.032 to 0.955)*** | 0.462(-0.032 to 0.955) | 0.250(0.050 to 1.280) | Fixed | 0.0000 | 0.067 | 0.067 | 0.426 | 0.300 to 17.302 | 0.0 | 0.867 | 0.991 | Yes | No | V | Low |
| IOE/ air-leak/blue-tinged saline^17^ | CRD | 222/3949 | 12 | 2 | 10 | ***0.494(0.362 to 0.676)*** | 0.510(0.367 to 0.710) | 0.318(0.145 to 0.695) | Fixed | 0.0000 | 0.000 | 0.000 | 0.004 | 0.856 to 6.072 | 0.0 | 0.575 | 0.880 | Yes | No | II | Low |
| IOFE^18^ | CRD | 59/1084 | 5 | 0 | 5 | ***0.375(0.208 to 0.676)*** | 0.387(0.210 to 0.715) | 0.318(0.145 to 0.695) | Fixed | 0.0000 | 0.001 | 0.002 | 0.004 | 0.856 to 6.072 | 0.0 | 0.482 | 0.749 | Yes | No | IV | Low |
| Seprafilm^56^ | gastrointestinal neoplasms | 40/1731 | 5 | 2 | 3 | *1.256(0.669 to 2.358)* | 1.238(0.645 to 2.374) | 0.694(0.170 to 2.833) | Fixed | 0.0000 | 0.478 | 0.521 | 0.610 | 0.393 to 13.230 | 0.0 | 0.655 | 0.265 | Yes | No | V | Low |
| Blood transfusion^7^ | RC | 286/18462 | 3 | 0 | 3 | 2.074(1.556 to 2.766) | ***3.178(1.217 to 8.294)*** | 1.531(1.081 to 2.168) | Random | 0.5410 | 0.000 | 0.018 | 0.016 | 0.357 to 14.555 | 81.3 | 0.005 | 0.282 | Yes | Yes | IV | Low |
| DC surgery^10^ | traumatic colonic injurie | 46/681 | 6 | 0 | 6 | ***4.426(2.238 to 8.751)*** | 4.779(1.778 to 12.846) | 0.511(0.052 to 5.003) | Fixed | 0.5283 | 0.000 | 0.002 | 0.564 | 0.079 to 65.907 | 42.8 | 0.137 | 0.264 | Yes | No | IV | Very Low |
| ICG^19^ | RC | 427/4738 | 22 | 3 | 19 | ***0.350(0.274 to 0.448)*** | 0.361(0.258 to 0.505) | 0.136(0.038 to 0.487) | Fixed | 0.1704 | 0.000 | 0.000 | 0.002 | 0.345 to 15.061 | 30.3 | 0.089 | 0.171 | No | No | I | Low |
| ICG (Grade A)^19^ | RC | 62/1206 | 6 | 1 | 5 | ***0.217(0.117 to 0.402)*** | 0.220(0.118 to 0.412) | 0.062(0.012 to 0.333) | Fixed | 0.0000 | 0.000 | 0.000 | 0.001 | 0.286 to 18.196 | 0.0 | 0.466 | 0.596 | Yes | No | II | Low |
| ICG (Grade B)^19^ | RC | 41/1206 | 6 | 1 | 5 | *0.693(0.364 to 1.319)* | 0.683(0.341 to 1.366) | 0.292(0.079 to 1.072) | Fixed | 0.0000 | 0.264 | 0.281 | 0.064 | 0.447 to 11636 | 0.0 | 0.493 | 0.423 | No | Yes | V | Low |
| ICG (Grade C)^19^ | RC | 32/1206 | 6 | 1 | 5 | *0.972(0.490 to 1.925)* | 1.044(0.492 to 2.213) | 0.588(0.137 to 2.528) | Fixed | 0.0315 | 0.934 | 0.912 | 0.475 | 0.350 to 14.840 | 3.4 | 0.395 | 0.813 | Yes | Yes | V | Low |
| ICG (Asian)^19^ | RC | 200/2540 | 7 | 0 | 7 | 0.306(0.209 to 0.448) | ***0.379(0.178 to 0.804)*** | 0.106(0.038 to 0.295) | Random | 0.6303 | 0.000 | 0.012 | 0.000 | 0.222 to 23.409 | 66.6 | 0.006 | 0.599 | Yes | Yes | IV | Low |
| ICG (Europe)^19^ | RC | 189/1672 | 11 | 2 | 9 | ***0.320(0.218 to 0.467)*** | 0.332(0.226 to 0.487) | 0.136(0.038 to 0.487) | Fixed | 0.0000 | 0.000 | 0.000 | 0.002 | 0.463 to 11.227 | 0.0 | 0.864 | 0.016 | No | Yes | II | Low |
| ICG (North America)^19^ | RC | 40/526 | 4 | 1 | 3 | *0.696(0.367 to 1.320)* | 0.733(0.378 to 1.421) | 0.944(0.456 to 1.955) | Fixed | 0.0000 | 0.267 | 0.357 | 0.878 | 0.918 to 5.663 | 0.0 | 0.401 | 0.002 | No | Yes | V | Low |
| Saelant^16^ | Gastrointestinal diseases | 230/3024 | 14 | 5 | 9 | ***0.374(0.271 to 0.517)*** | 0.394(0.283 to 0.547) | 0.197(0.066 to 0.583) | Fixed | 0.0000 | 0.000 | 0.000 | 0.003 | 0.584 to 8.897 | 0.0 | 0.835 | 0.038 | No | Yes | II | Low |
| Omentoplasty^57^ | CRD | 63/943 | 3 | 0 | 3 | 0.550(0.323 to 0.937) | *0.450(0.165 to 1.226)* | 0.182(0.049 to 0.668) | Random | 0.4708 | 0.028 | 0.118 | 0.010 | 0.219 to 23.752 | 59.9 | 0.082 | 0.272 | No | Yes | V | Very Low |
| **Preoperative preparation** | |  |  |  |  |  |  |  |  |  |  |  |  |  |  |  |  |  |  |  |  |
| High surgeon volume^59^ | RC | 139/2202 | 3 | 0 | 3 | *0.702(0.474 to 1.041)* | 0.669(0.383 to 1.172) | 0.264(0.082 to 0.852) | Fixed | 0.1039 | 0.078 | 0.160 | 0.026 | 0.432 to 12.022 | 42.7 | 0.175 | 0.208 | Yes | Yes | V | Low |
| OMBP (all)^60^ | CRD | 250/5311 | 16 | 16 | 0 | *1.062(0.824 to 1.369)* | 1.209(0.801 to 1.824) | 0.418(0.170 to 1.027) | Fiexd | 0.2966 | 0.644 | 0.367 | 0.057 | 0.398 to 13.051 | 48.3 | 0.016 | 0.013 | Yes | Yes | V | Low |
| OMBP (vs enema)^60^ | CRD | 45/985 | 4 | 4 | 0 | 1.434(0.804 to 2.557) | *2.435(0.452 to 13.121)* | 0.417(0.106 to 1.644) | Random | 2.3349 | 0.222 | 0.300 | 0.211 | 0.037 to 139.957 | 82.9 | 0.001 | 0.222 | Yes | Yes | V | Low |
| MBP (vs rectal enema)^62^ | CRC | 255/5113 | 12 | 12 | 0 | *0.926(0.715 to 1.200)* | 0.916(0.702 to 1.195) | 0.829(0.560 to 1.228) | Fiexd | 0.0000 | 0.561 | 0.518 | 0.350 | 1.396 to 3.724 | 0.0 | 0.573 | 0.712 | Yes | Yes | V | Low |
| MBP^62^ | CRC | 1152/16036 | 21 | 21 | 0 | ***0.826(0.729 to 0.935)*** | 0.981(0.777 to 1.237) | 0.667(0.555 to 0.802) | Fiexd | 0.0769 | 0.003 | 0.869 | 0.000 | 1.113 to 4.672 | 42.2 | 0.025 | 0.007 | Yes | Yes | V | Low |
| PEG +AB (vs AB)^63^ | CRD | 20/482 | 4 | 4 | 0 | *0.948(0.386 to 2.332)* | 0.974(0.378 to 2.511) | 0.292(0.029 to 2.933) | Fiexd | 0.0000 | 0.908 | 0.956 | 0.295 | 0.127 to 40.833 | 0.0 | 0.602 | 0.148 | No | Yes | V | Low |
| PEG+enema+AB (vs AB)^63^ | CRD | 24/487 | 3 | 2 | 1 | *1.305(0.569 to 2.997)* | 1.293(0.558 to 2.996) | 1.014(0.299 to 3.445) | Fiexd | 0.0000 | 0.530 | 0.550 | 0.982 | 0.495 to 10.505 | 0.0 | 0.724 | 0.940 | No | No | V | Low |
| Fuid diet for 3 days+AB (vs AB) ^63^ | CRD | 16/94 | 1 | 1 | 0 | 0.950(0.324 to 2.786) | 0.950(0.324 to 2.786) | 0.950(0.324 to 2.786) | NA | NA | 0.926 | 0.926 | 0.926 | NA | NA | NA | NA | NA | NA | V | Low |
| MBP+OAB (vs MBP)^64^ | CRD | 3425/47610 | 35 | 26 | 9 | ***0.454(0.421 to 0.490)*** | 0.468(0.408 to 0.536) | 0.477(0.426 to 0.533) | Fiexd | 0.0244 | 0.000 | 0.000 | 0.000 | 1.517 to 3.427 | 25.4 | 0.092 | 0.668 | Yes | Yes | II | Low |
| MBP+OAB (vs OAB)^64^ | CRD | 437/19360 | 3 | 2 | 1 | *0.789(0.593 to 1.051)* | 0.786(0.586 to 1.052) | 0.752(0.561 to 1.010) | Fiexd | 0.0013 | 0.105 | 0.106 | 0.058 | 1.563 to 3.327 | 0.4 | 0.366 | 0.710 | Yes | No | V | Low |
| Oral+IV(vs IV)^65^ | CRD | 25/804 | 9 | 0 | 9 | *0.610(0.281 to 1.327)* | 0.613(0.265 to 1.415) | 0.297(0.030 to 2.969) | Fiexd | 0.0000 | 0.213 | 0.251 | 0.301 | 0.129 to 40.282 | 0.0 | 0.892 | 0.079 | No | Yes | V | Low |
| Oral fluoroquinolone AB+IV(vs IV)^66^ | CRD | 13/1026 | 3 | 3 | 0 | ***0.200(0.050 to 0.795)*** | 0.211(0.052 to 0.853) | 0.258(0.053 to 1.261) | Fiexd | 0.0000 | 0.022 | 0.029 | NA | 0.315 to 16.528 | 0.0 | 0.599 | NA | Yes | NA | IV | Low |
| Glucocorticoids^67^ | CRD | 9/163 | 4 | 4 | 0 | *1.253(0.348 to 4.503)* | 1.248(0.311 to 5.007) | 1.000(0.130 to 7.691) | Fiexd | 0.0000 | 0.730 | 0.755 | NA | 0.178 to 29.205 | 0.0 | 0.460 | 0.618 | Yes | NA | V | Very Low |
| pRCT^61^ | RC | 89/828 | 2 | 2 | 0 | *0.984(0.633 to 1.530)* | 0.984(0.633 to 1.531) | 1.015(0.636 to 1.620) | Fiexd | 0.0000 | 0.943 | 0.944 | 0.949 | 1.271 to 4.090 | 0.0 | 0.691 | NA | No | NA | V | Low |
| pRT^61^ | RC | 206/2697 | 6 | 6 | 0 | *1.042(0.782 to 1.388)* | 1.042(0.779 to 1.393) | 0.774(0.444 to 1.350) | Fiexd | 0.0000 | 0.779 | 0.781 | 0.366 | 1.138 to 4.569 | 0.0 | 0.474 | 0.782 | No | Yes | V | Low |
| nCRT^69^ | RC | 464/4856 | 13 | 2 | 11 | *1.067(0.870 to 1.309)* | 1.059(0.861 to 1.304) | 0.975(0.739 to 1.286) | Fiexd | 0.0000 | 0.534 | 0.587 | 0.858 | 1.613 to 3.223 | 0.0 | 0.504 | 0.148 | Yes | No | V | Low |
| nCRT<8w^69^ | RC | 41/750 | 7 | 0 | 7 | *1.246(0.655 to 2.370)* | 1.112(0.555 to 2.231) | 0.531(0.159 to 1.777) | Fiexd | 0.0000 | 0.502 | 0.764 | 0.304 | 0.504 to 10.307 | 0.0 | 0.526 | 0.006 | Yes | Yes | V | Low |
| nRT^70^ | CRC | 2120/23453 | 59 | 10 | 49 | ***1.164(1.057 to 1.282)*** | 1.170(1.039 to 1.319) | 0.975(0.739 to 1.286) | Fiexd | 0.0267 | 0.002 | 0.010 | 0.858 | 1.343 to 3.871 | 14.7 | 0.174 | 0.617 | Yes | No | IV | Low |
| Long-course nRT^70^ | CRC | 1111/13715 | 39 | 0 | 39 | ***1.189(1.045 to 1.353)*** | 1.201(1.028 to 1.403) | 0.946(0.605 to 1.479) | Fiexd | 0.0185 | 0.009 | 0.021 | 0.807 | 1.190 to 4.370 | 8.7 | 0.317 | 0.412 | Yes | No | IV | Low |
| Long-course nRT＜8W^70^ | CRC | 772/9509 | 28 | 0 | 28 | *1.043(0.892 to 1.220)* | 1.084(0.854 to 1.375) | 0.975(0.739 to 1.286) | Fiexd | 0.0798 | 0.596 | 0.508 | 0.858 | 1.052 to 4.943 | 27.1 | 0.094 | 0.294 | Yes | No | V | Low |
| Short-course nRT^70^ | CRC | 647/6550 | 11 | 0 | 11 | ***1.239(1.036 to 1.483)*** | 1.248(1.016 to 1.534) | 0.918(0.610 to 1.380) | Fiexd | 0.0139 | 0.019 | 0.035 | 0.679 | 1.269 to 4.098 | 11.5 | 0.335 | 0.796 | No | No | IV | Low |
| nCT^4^ | CRC | 545/15610 | 5 | 0 | 5 | ***2.191(1.394 to 3.444)*** | 2.288(1.119 to 4.678) | 0.877(0.277 to 2.774) | Fiexd | 0.2935 | 0.001 | 0.023 | 0.823 | 0.322 to 16.161 | 46.2 | 0.115 | 0.414 | Yes | No | IV | Low |
| Stent^71^ | Left colorectal cancer with malignant  obstruction | 28/515 | 8 | 8 | 0 | *1.123(0.547 to 2.304)* | 1.274(0.520 to 3.124) | 0.068(0.003 to 1.411) | Fiexd | 0.1553 | 0.752 | 0.596 | 0.082 | 0.043 to 120.231 | 9.2 | 0.359 | 0.248 | Yes | Yes | V | Low |
| **Postoperative management** |  |  |  |  |  |  |  |  |  |  |  |  |  |  |  |  |  |  |  |  |  |
| Oral + IV AB (vs IV AB)^72^ | colorectal neoplasm | 69/1768 | 5 | 5 | 0 | ***0.547(0.330 to 0.906)*** | 0.559(0.335 to 0.932) | 0.610(0.304 to 1.224) | Fiexd | 0.0000 | 0.019 | 0.026 | 0.164 | 0.955 to 5.445 | 0.0 | 0.748 | 0.435 | No | No | IV | Low |
| Rapid rehabilitation^73^ | gastrointestinal diseases | 180/3378 | 19 | NR | NR | *0.896(0.658 to 1.221)* | 0.889(0.648 to 1.221) | 0.728(0.392 to 1.352) | Fiexd | 0.0000 | 0.487 | 0.469 | 0.315 | 1.052 to 4.943 | 0.0 | 0.990 | 0.789 | Yes | Yes | V | Low |
| ICWH^74^ | CRC | 41/1499 | 4 | 2 | 2 | ***2.049(1.056 to 3.976)*** | 1.751(0.692 to 4.430) | 1.009(0.250 to 4.067) | Fiexd | 0.3606 | 0.034 | 0.237 | 0.990 | 0.233 to 22.315 | 40.7 | 0.168 | 0.235 | No | No | V | Low |
| Selective COX-2 NSAIDs^21^ | gastrointestinal diseases | 416/4404 | 8 | 2 | 6 | 1.321(1.010 to 1.727) | *1.675(0.896 to 3.130)* | 0.908(0.614 to 1.343) | Random | 0.4219 | 0.042 | 0.106 | 0.630 | 0.431 to 12.050 | 66.5 | 0.004 | 0.394 | Yes | No | V | Low |
| NSAIDs^21^ | gastrointestinal diseases | 1922/31876 | 24 | 6 | 18 | 1.262(1.142 to 1.396) | ***1.688(1.278 to 2.230)*** | 1.145(0.946 to 1.385) | Random | 0.2635 | 0.000 | 0.000 | 0.164 | 0.634 to 8.200 | 79.1 | 0.000 | 0.027 | No | Yes | III | Low |
| Non-systematic NSAIDs^21^ | gastrointestinal diseases | 1831/29958 | 13 | 0 | 13 | 1.174(1.058 to 1.302) | ***1.344(1.033 to 1.749)*** | 0.621(0.472 to 0.816) | Random | 0.1738 | 0.003 | 0.028 | 0.001 | 0.776 to 6.695 | 81.1 | 0.000 | 0.173 | Yes | Yes | IV | Low |
| Non-selective NSAIDs^75^ | gastrointestinal diseases | 112/1466 | 8 | 5 | 3 | ***2.964(1.990 to 4.415)*** | 3.385(1.688 to 6.789) | 2.037(1.211 to 3.427) | Fiexd | 0.2870 | 0.000 | 0.001 | 0.007 | 0.527 to 9.864 | 37.6 | 0.142 | 0.570 | Yes | No | IV | Low |
| Selective NSAIDs^75^ | gastrointestinal diseases | 76/1264 | 4 | 2 | 2 | 2.574(1.547 to 4.282) | *2.269(0.682 to 7.556)* | 1.175(0.504 to 2.741) | Random | 0.8978 | 0.000 | 0.182 | 0.709 | 0.177 to 29.239 | 69.1 | 0.021 | 0.757 | No | No | V | Low |
| Diclofenac^76^ | CRD | 194/2866 | 3 | 0 | 3 | ***2.787(1.962 to 3.960)*** | 2.744(1.941 to 3.880) | 2.752(1.770 to 4.278) | Fiexd | 0.0000 | 0.000 | 0.000 | 0.000 | 1.313 to 3.958 | 0.0 | 0.418 | 0.298 | No | Yes | II | Low |
| Ketorolac^77^ | CRC | 11/435 | 2 | 0 | 2 | *1.912(0.559 to 6.541)* | 2.109(0.276 to 16.139) | 1.047(0.245 to 4.486) | Fiexd | 1.0396 | 0.302 | 0.472 | 0.950 | 0.104 to 50.061 | 42.1 | 0.189 | NA | Yes | NA | V | Low |
| Probiotics^78^ | CRC | NR | 8 | 7 | 1 | ***0.256(-0.173 to 0.685)*** | 0.256(-0.173 to 0.685) | 0.130(0.010 to 0.990) | Fiexd | 0.0000 | 0.242 | 0.242 | 0.603 | 0.417 to 12.469 | 0.0 | 0.989 | 0.781 | Yes | No | V | Low |
| Early feeding^79^ | gastrointestinal diseases | 29/1075 | 13 | 13 | 0 | *0.709(0.350 to 1.438)* | 0.694(0.328 to 1.467) | 0.475(0.086 to 2.630) | Fiexd | 0.0000 | 0.340 | 0.339 | 0.394 | 0.269 to 19.335 | 0.0 | 0.903 | 0.211 | Yes | No | V | Low |

**Abbreviations:** ASA, American Society of Anaesthesiologist; BMI, body mass index; ALT, air leak test; LCA, left colic artery; IMA, inferior mesenteric artery; TDT, transanal drainage tube; CRD, colorectal diseases; SFM, splenic fexure mobilization; HRR, high rectal resection; LRR, low rectal resection; LT, laparotomic; LS, laparoscopic; IORT, intraoperative radiotherapy; EBRT, external beam radiotherapy; IOE, intraoperative endoscopy; IOFE, intra-operative flexible endoscopy; Seprafilm, hyaluronate-carboxy-methylcellulose-based membrane; DC, damage control; ICG, indocyanine green; LI, loop ileostomy; OMBP, oral mechanical bowel preparation; pRCT, preoperative chemoradiotherapy; pRT, preoperative radiotherapy; MBP, mechanical bowel preparation; PEG, polyethylene glycol; AB, antibiotics; OAB, oral antibiotics; IV, intravenous; GCs, glucocorticoids; nCRT, neoadjuvant chemoradiotherapy; nRT, neoadjuvant radiotherapy; nCT, neoadjuvant chemotherapy; ICWH, intraperitoneal chemotherapy without hyperthermia; NSAIDs, non-steroidal anti-inflammatory drugs; CRC, colorectal cancer; CRD, colorectal diseases; RC, rectal cancer; NR, not reported; NA, not available; RCT, randomized controlled study; OE, observational study; AMSTAR 2, a measurement tool to assess systematic reviews; CI, confidence interval.
